# Carotenoid biosynthesis genes *LcLCYB*, *LcLCYE*, and *LcBCH* from wolfberry confer increased carotenoid content and improved salt tolerance in tobacco

**DOI:** 10.1038/s41598-024-60848-3

**Published:** 2024-05-08

**Authors:** Chen Li, Caili Wang, Zhiyang Cheng, Yu Li, Wenjing Li

**Affiliations:** College of Life Sciences, Dezhou University, 566 University Road, Dezhou, 253023 Shandong Province China

**Keywords:** Abiotic, Secondary metabolism

## Abstract

Carotenoids play essential roles in plant growth and development and provide plants with a tolerance to a series of abiotic stresses. In this study, the function and biological significance of lycopene β-cyclase, lycopene ε-cyclase, and β-carotene hydroxylase, which are responsible for the modification of the tetraterpene skeleton procedure, were isolated from *Lycium chinense* and analyzed. The overexpression of *lycopene β-cyclase*, *lycopene ε-cyclase*, and *β-carotene hydroxylase* promoted the accumulation of total carotenoids and photosynthesis enhancement, reactive oxygen species scavenging activity, and proline content of tobacco seedlings after exposure to the salt stress. Furthermore, the expression of the carotenoid biosynthesis genes and stress-related genes (*ascorbate peroxidase*, *catalase*, *peroxidase*, *superoxide dismutase*, and *pyrroline-5-carboxylate reductase*) were detected and showed increased gene expression level, which were strongly associated with the carotenoid content and reactive oxygen species scavenging activity. After exposure to salt stress, the endogenous abscisic acid content was significantly increased and much higher than those in control plants. This research contributes to the development of new breeding aimed at obtaining stronger salt tolerance plants with increased total carotenoids and vitamin A content.

## Introduction

Plants face numerous changing environmental stresses, including biotic and abiotic. Salt stress is among the most significant abiotic factors to modern agriculture, affecting plant geographical distribution and limiting agricultural production and productivity^1,2^. Nowadays, more than 4.4% of topsoil and more than 8.7% of sub topsoil of the total land was reported as salt affected, covering 73% of the global land area^3^. When plants are exposed to salinized soil, excess ions are absorbed from the environment, resulting in insufficient water uptake. The accumulated sodium ions would cause many adverse effects on living plants, including osmotic stress, high sodium toxic stress, and ion-induced oxidative stress^4,5^. It leads to the disruption of physiological processes and inhibits the growth and development of plants^4^. Oxidative stress is generated by the increased accumulation of reactive oxygen species (ROS)^6^, including hydrogen peroxide (H_2_O_2_), hydroxyl radical (•OH), and superoxide anions (O_2_^−^), which are highly reactive in plants^7^. Excessive ROS accumulation has been demonstrated to be harmful to the cell components by inducing abnormal oxidations of proteins, lipids, and nucleic acids, resulting in the malfunction of key cellular organelles and disruption of pigment synthesis^8^, photosystem (PS) I, PS II^9^, enzyme activity, and secondary metabolite accumulation^10^. To survive in the salinized environment, a series of strategies have been evolved in plants. Improving oxidative stress tolerance on physiological, biochemical, and gene expression levels is a promising approach of genetic modification to enhance plants' salt tolerance^4^.

Carotenoids, a group of more than 600 isoprenoid pigments, are synthesized in chloroplasts and chromoplasts of plants, the photosynthetic algae, bacteria, and fungi^11,12^. In higher plants, carotenoids have series of essential functions, such as structural and accessory pigments in the photosynthetic complex, photoprotectors against oxidative stress damage, and precursors of some specific plant hormones^13,14^. In humans and animals, carotenoids act as antioxidants in the body's immune system^15^ and provide nutrition as both precursors of vitamin A and retinoid compounds^14^. Due to works in *Arabidopsis*, maize, rice, and some ornamental plants^16,17^, the carotenoid biosynthesis pathway has been well elucidated and generally divided into three consequential stages. Briefly, the first stage was initiated through the catalyzation of isopentenyl pyrophosphate isomerase (IPI), geranylgeranyl pyrophosphate synthase (GGPS), and phytoene synthase (PSY), transformed four molecules of the isopentenyl diphosphate (IPP) and dimethylallyl diphosphate (DMAPP) into the 15-cis-phytoene, which were usually fast transformed and thus not accumulated in tissue^18,19^. The desaturation of the tetraterpene skeleton was subsequently accomplished through four desaturation steps by phytoene desaturase (PDS), carotene isomerase (CRTISO), and ζ-carotene desaturase (ZDS), and the lycopene was formed^20,21^. The third stage of tetraterpene skeleton modification was separated into two branches, for one branch (β-ε-) was catalyzed by β-cyclase (LCYB) and ε-cyclase (LCYE), and the a-carotene was formed. The other pathway (β-β-) was first catalyzed by LCYB and formed the β-carotene^22,23^. The α-carotene and β-carotene are the main forms of provitamin A, which could be transformed to activated form of vitamin A. Vitamin A deficiency leads to visual impairment and increase the incidence and severity of infectious diseases^24,25^. Then, the α-carotene and β-carotene were catalyzed by β-carotene hydroxylase (BCH), through β-ε- and β-β- branches, respectively, and lutein and zeaxanthin were obtained, which act as non-provitamin A carotenoids and are beneficial for human health^14,23,26^. The followed xanthophyll cycle through the β-β- branch was regulated by the light intensity and pH in the thylakoids, which were catalyzed by violaxanthin de-epoxidase (VDE) and zeaxanthin epoxidase (ZEP). Under a strong light intensity, improved activity of VDE and a higher content of zeaxanthin were detected with a decreased pH in the thylakoid. Meanwhile, the activity of ZEP and the content of violaxanthin would increase with a higher pH under a weak light intensity environment^27^.

Currently known, the genes involved in the carotenoid biosynthesis pathway have been isolated, identified, and found to be closely related to plant growth and development process. As reported, the silenced *NtPDS* by RNAi resulted in a deficiency of carotenoid accumulation, albinism of tissues, and even death of the tobacco seedlings^28^. The expression level of *ZDS* has been reported to be well paralleled with the formation of carotenoids in leaves, inflorescences, and developing fruits^29^. In addition, some studies showed that the overexpression of carotenoid biosynthesis genes significantly increased the carotenoid content and improved the tolerance to abiotic stresses. The overexpression of *IbGGPS* or *IbZDS* significantly increased the carotenoid content and improved the tolerance to the osmotic stress in transgenic plants^29,30^. Our previous work also demonstrated that overexpression of wolfberry *PDS*, *ZDS*, or *CRTISO* significantly increased the carotenoid accumulation and salt tolerance in the transgenic tobaccos, and there was a close correlation between the carotenoid content and the salt tolerance^31^.

In the future, crops with improved tolerance to salt stress will be required to guarantee food security. The objectives of our study were to identify the robust stress-related genes which could be used in the pre-breeding work for the generation of stress-tolerant elite crop lines. In this work, genes encoding LCYB, LCYE, and BCH, which are responsible for the modification of the tetraterpene skeleton process, were selected for their biological significance and cloned from the ripened red fruits of wolfberry, which were widely spread in the area of slight salinization and rich in the carotenoids. Biological functions of *LcLCYB*, *LcLCYE*, and *LcBCH* were identified in *Nicotiana tabacum*, a model system for investigating novel genes in salt tolerance, and found overexpression of *LcLCYB*, *LcLCYE*, or *LcBCH* significantly increased the carotenoid content and enhanced salt tolerance in the seedlings of transgenic tobacco. In addition, this study also analyzed the underlying molecular mechanism for constitutive accumulation of carotenoids in transgenic plants under salt stress.

## Results

### Phylogenetic analysis and multiple sequence alignments of *LCYB*, *LCYE*, and *BCH* genes

Three wolfberry carotenoid biosynthesis pathway members responsible for the modification of the tetraterpene skeleton, LcLCYB, LcLCYE, and LcBCH, were designated based on BLAST results of the protein sequences of genes from *Arabidopsis thaliana*, *Nicotiana tabacum*, *Oryza sativa*, *Capsicum annuum*, and *Solanum lycopersicum*. These three genes were cloned from the cDNA of the ripened red fruits of *L. chinense* (Figure S1). General analyses about the protein's physical and chemical properties and predicted subcellular localizations were listed in Table S1. Multiple sequence alignments showed that the *LcLCYB*, *LcLCYE*, and *LcBCH* coding proteins had high similarity with predicted proteins in *C. annuum* (92.37, 93.24, and 75.16%), *Solanum tuberosum* (92.8, 92.93, and 85.9%), *A. thaliana* (80.32, 73.35, and 68.04%), *Zea mays* (69.58, 67.19, and 59.46%), *N. tabacum* (90.8, 93.69, and 74.67%), and *O. sativa* (68.75, 67.51, and 69.34%), respectively. The consensus sequences of these proteins were shown in Figure S2–S4.

To investigate the evolutionary relationships of LCYB, LCYE, and BCH proteins, separate unrooted phylogenic trees were constructed by the neighbor-joining method, revealing that the wolfberry *LCYB*, *LCYE*, and *BCH* coding proteins were closely related with their corresponding homogenous genes in Arabidopsis, rice, tobacco, maize and some *Solanaceae* species (Figure S5). It was proposed that proteins responsible for specifically catalytic function were highly conserved in plants. Thus, detailed information about protein structures of LCYB, LCYE, and BCH was obtained and demonstrated that the desaturase domain and FAD-binding domains were well conserved, as shown in Figure S6. To test whether LcLCYB, LcLCYE, and LcBCH had their corresponding catalytic function, the complementation expression assay was done in vitro, respectively, demonstrating their expression could fulfill the catalytic process (Figure S7).

### Overexpression of *LcLCYB*, *LcLCYE*, and *LcBCH* promoted carotenoids accumulation in tobacco seedlings to varying degrees

**Figure 1:**
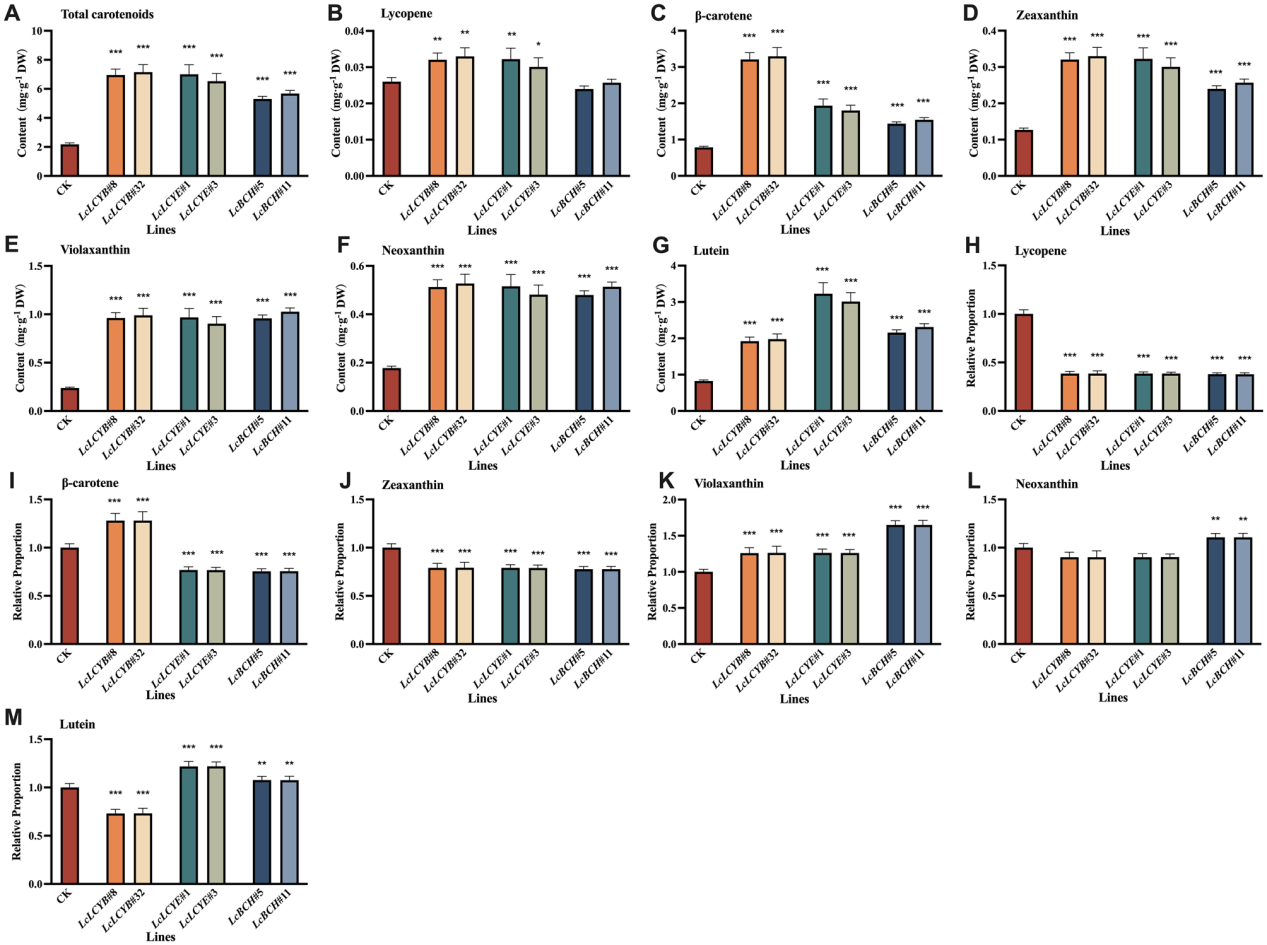
Detection of carotenoid content in tobacco seedlings with high performance liquid chromatography. Content of (**A**) total carotenoids, (**B**) lycopene, (**C**) β-carotene, (**D**) zeaxanthin, (**E**) violaxanthin, (**F**) neoxanthin, and (**G**) lutein; relative proportion of (**H**) lycopene, (**I**) β-carotene, (**J**) zeaxanthin, (**K**) violaxanthin, (**L**) neoxanthin, and (**M**) lutein were analyzed in freeze-dried samples. Data were obtained from three independent experiments, and analysis of variance were performed. The results were presented as means ± standard deviation. *P* values of < 0.05 (*), < 0.01 (**) and < 0.001 (***) were considered to be significant statistically.

To elucidate the function of *LcLCYB*, *LcLCYE*, and *LcBCH*, more than 50 independent tobacco overexpression lines were obtained for each gene and named as *Gene#Number*. The results showed that each gene was expressed in its overexpression transgenic lines but not in the wild-type plants (Figure S8). Compared with the CK plants, total carotenoid content in *LcLCYB*, *LcLCYE*, and *LcBCH* overexpression lines were much higher and highly correlated with its exogenous gene expression level (Figure S9). Homozygous seeds generated from the overexpression lines, *LcLCYB*#8, *LcLCYB*#32, *LcLCYE*#1, *LcLCYE#*3, and *LcBCH*#5, *LcBCH*#11, which showed the highest expression level and total carotenoid content in each genotype, were used for further experiments. Among these overexpression lines, *35S:LcLCYB*, *35S:LcLCYE*, and *35S:LcBCH* showed higher total carotenoid content, which was about 3, 3, and 2.5-fold of the CK plants, respectively, while there was no significant difference between *35S:LcLCYB* and *35S:LcLCYE* transgenic tobaccos with genes at a similar gene expression level (Figure S8 and S9). Each component of carotenoids was then examined in the *35S:LcLCYB*, *35S:LcLCYE*, and *35S:LcBCH* overexpression lines by  high performance liquid chromatography (HPLC), and found the content of each detected pigment increased significantly in leaves, except the lycopene in *35S:LcBCH* ones (Fig. 1). In addition, the proportion of most pigments accumulating in the overexpressing tobacco leaves varied significantly different. For most carotenoid components, the *35S:LcLCYB* and *35S:LcLCYE* tobaccos exhibited a similar proportion, except the β-carotene and lutein. As the downstream catalytic protein of LCYE in the carotenoid biosynthesis pathway, the proportion of each pigment in *35S:LcBCH* correlated well with *35S:LcLCYE*, except neoxanthin (Fig. 1).

### Overexpression of *LcLCYB*, *LcLCYE*, and *LcBCH* enhanced tobacco salt tolerance by improving photosynthesis

**Figure 2:**
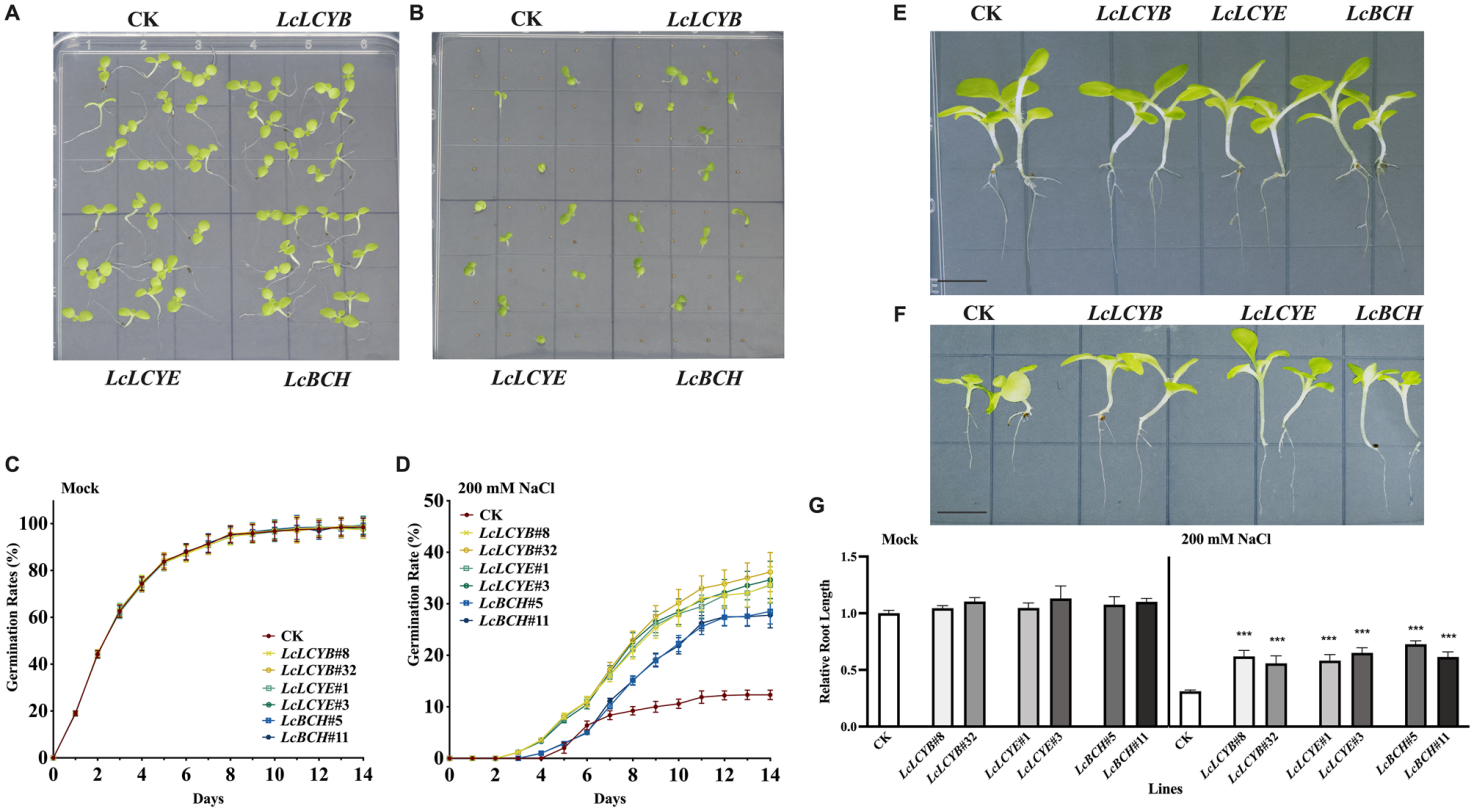
Effects of salt tolerance analysis in tobacco seedlings. (**A**) Tobacco seeds germinated in 1/2 Murashige and Skoog (MS) and (**B**) 1/2 MS with 200 mM NaCl of 14th day after germination. Germination rates of seeds were calculated in (**C**) 1/2 MS, and (**D**) 1/2 MS with 200 mM NaCl, respectively. Seedlings in (**E**) absence, and (**F**) presence of salt stress, and (**G**) the lengths of roots. Scale bars = 1 cm. Seed germination rate was defined as the number of seeds obvious emergence of the radicle through the seed coat per 100 seeds. 1/2 MS medium supplied with 200 mM NaCl was employed for the salt treatment while 1/2 MS medium was set as mock-treated. Data were obtained from three independent experiments, and analysis of variance were performed. The results were presented as means ± standard deviation. *P* values of < 0.001 (***) were considered to be significant statistically.

For further characterizations of gene functions, phenotypic analysis of transgenic tobaccos was applied. 1/2 Murashige and Skoog (MS) medium supplied with 200 mM NaCl was employed for the salt treatment while 1/2 MS medium was set as mock-treated. After a 3-d 4 °C vernalization, the sterilized seeds of each genotype were sown on the 1/2 MS medium, and all the tobacco seeds showed a similar germination rate (Fig. 2A, C). Under 200 mM NaCl treatments, the seed germination process was significantly inhibited and delayed (Fig. 2B). The results showed that the initiation of seed germination in *35S:LcLCYB*, *35S:LcLCYE*, and *35S:LcBCH* was at the 3rd day, 1 day ahead of the CK plants (Fig. 2D). Furthermore, the *35S:LcLCYB*, *35S:LcLCYE*, and *35S:LcBCH* tobacco seeds demonstrated a significantly higher germination percentage from day 3, and was approximately 4, 4, and threefold of the CK plants on the 14th day (Fig. 2D). Subsequently, the 7-day old seedlings were transferred to the 1/2 MS medium supplemented with 200 mM NaCl for further phenotypic analysis, which demonstrated that exposure to NaCl treatments inhibited the growth of seedlings at different levels with a smaller leaf area and shorter primary root length of the CK plants (Fig. 2E–G).

**Figure 3:**
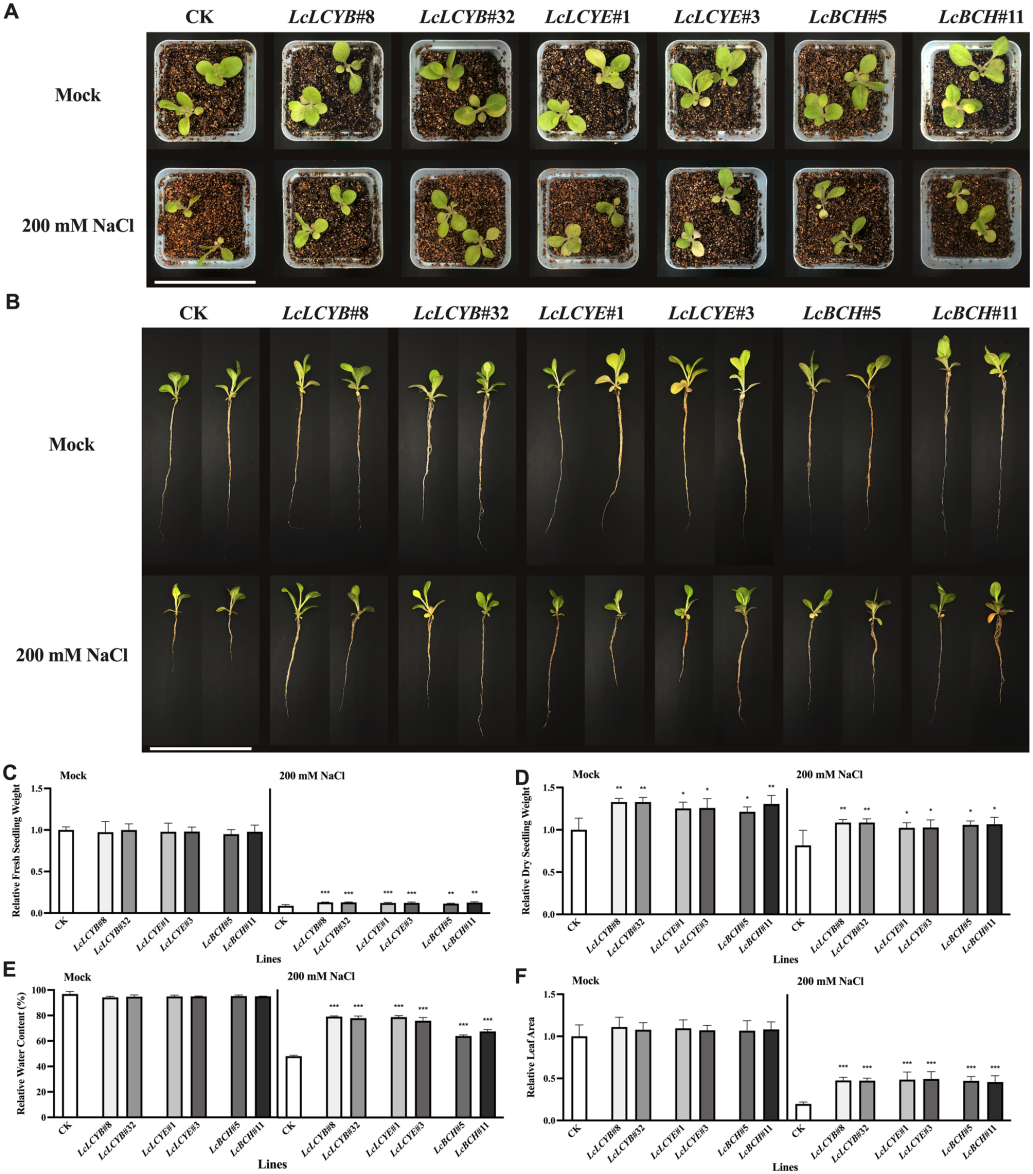
Effects of salt stress in tobacco seedlings. (**A, B**) Phenotypes of transgenic tobacco lines under salt stress for 14 days. Scale bars = 10 cm. (**C**) Relative water content, (**D**) relative fresh seedlings weight, (**E**) relative dry seedlings weight, and (**F**) relative leaf area of the seedlings. Seedlings treated. Data were obtained from three independent experiments, and analysis of variance were performed. The results were presented as means ± standard deviation. *P* values of < 0.05 (*), < 0.01 (**) and < 0.001 (***) were considered to be significant statistically. 200 mM NaCl labelled samples were collected from the seedlings treated with 200 mM NaCl for 2 weeks while *Mock* labelled were collected from fresh water treated seedlings.

**Figure 4:**
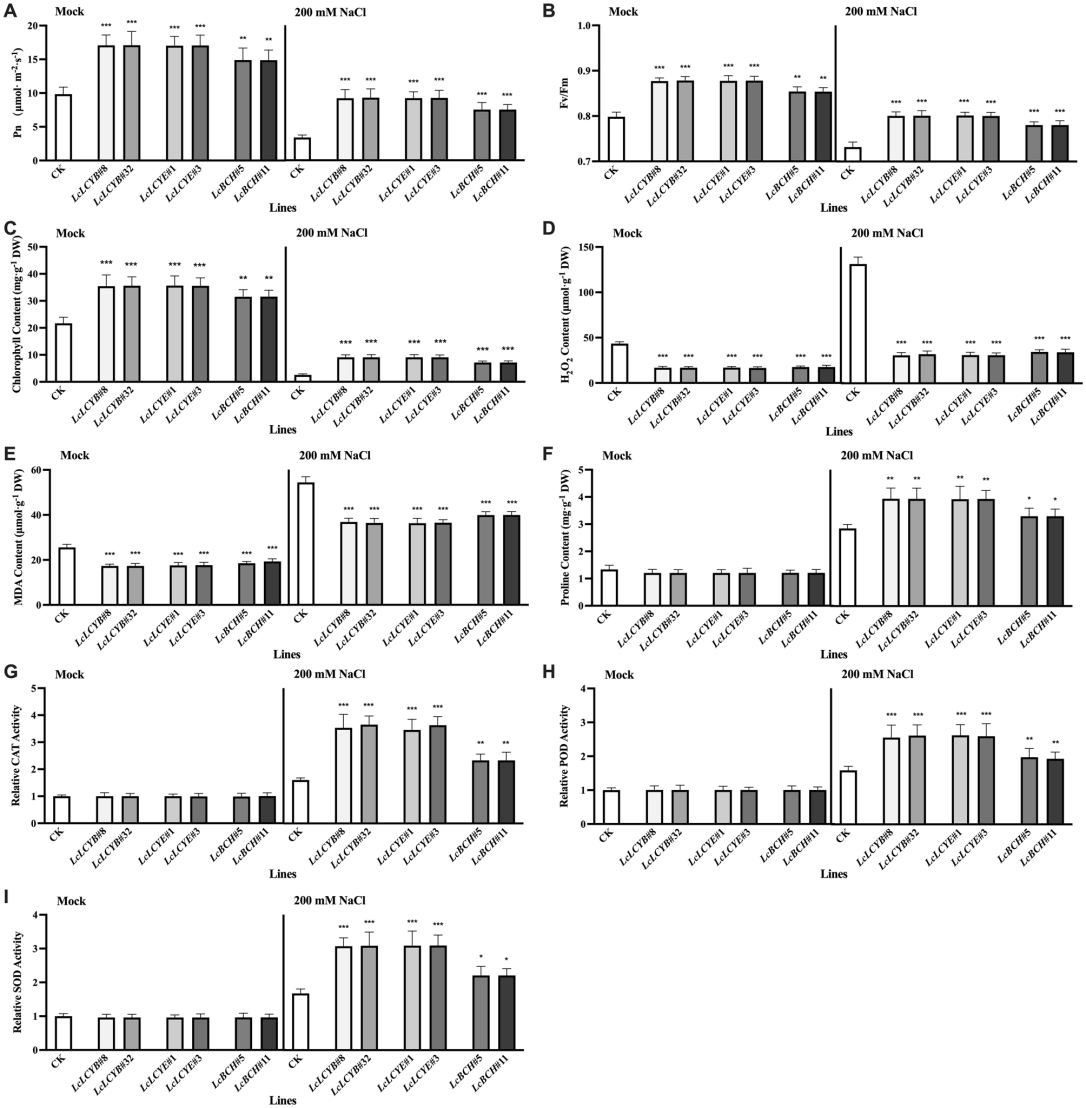
Effects of salt stress on photosynthetic and oxidative damage parameters in tobacco seedlings. (**A**) Net photosynthesis rates (Pn), (**B**) maximum quantum efficiency of PS II (Fv/Fm), and (**C**) chlorophyll content. Contents of (**D**) H_2_O_2_, (**E**) malondialdehyde (MDA), and (**F**) proline, and activities of (**G**) catalase (CAT), (**H**) peroxidase (POD), and (**I**) superoxide dismutase (SOD). Data were obtained from three independent experiments, and analysis of variance were performed. The results were presented as means ± standard deviation. *P* values of < 0.01 (**) and < 0.001 (***) were considered to be significant statistically. 200 mM NaCl labelled samples were collected from the seedlings treated with 200 mM NaCl for 2 weeks while *Mock* labelled were collected from fresh water treated seedlings.

Salt stress on soil-grown plants was applied by using tobacco plants treated with 200 mM NaCl solution, while fresh water was introduced as mock-treated. Phenotypes of the soil-grown plants treated with 200 mM NaCl solution were similar to the plate-grown seedlings, with shorter primary root length, more water loss, less seedling fresh and dry weight of the CK plants (Fig. 3). After 2 weeks' treatments, most plants turn yellow, indicating a large chlorophyll content reduction (Fig. 3B). By contrast with the CK plants, *35S:LcLCYB*, *35S:LcLCYE*, and *35S:LcBCH* transgenic plants showed a higher photosynthetic rate (Pn), maximal photochemical efficiency of PS II (Fv/Fm), and chlorophyll content, implying much more effective photosynthesis in these overexpression lines under NaCl treatments (Fig. 4). All the results above suggested that overexpression of *LcLCYB*, *LcLCYE*, and *LcBCH*, respectively, improved photosynthesis and helped transgenic tobaccos performed better under NaCl treatments.

### Relationship between carotenoid content, ROS scavenging, and proline accumulation in tobaccos under NaCl treatments

NaCl treatments resulted in strong oxidative stresses, the ROS scavenging activity, and proline accumulation were significantly increased in tobaccos (Fig. 4D–F). Compared with the CK plants, *35S:LcLCYB*, *35S:LcLCYE*, and *35S:LcBCH* tobacco seedlings maintained a much lower level of H_2_O_2_ and MDA, indicating a stronger ROS scavenging activity of transgenic plants before and after NaCl treatments. Meanwhile, increased proline accumulation was observed as well, which helped plants acclimatize multiple abiotic stresses, and showed a significantly higher content of *35S:LcLCYB*, *35S:LcLCYE*, and *35S:LcBCH* tobacco seedlings after exposure to NaCl treatments (Fig. 4F). In addition, results showed that activities of CAT, POD, and SOD, which are responsible for ROS scavenging in *LcLCYB*, *LcLCYE*, and *LcBCH* overexpression tobaccos, were significantly higher than the CK, also indicating a stronger antioxidant capacity (Fig. 4G–I).

**Figure 5:**
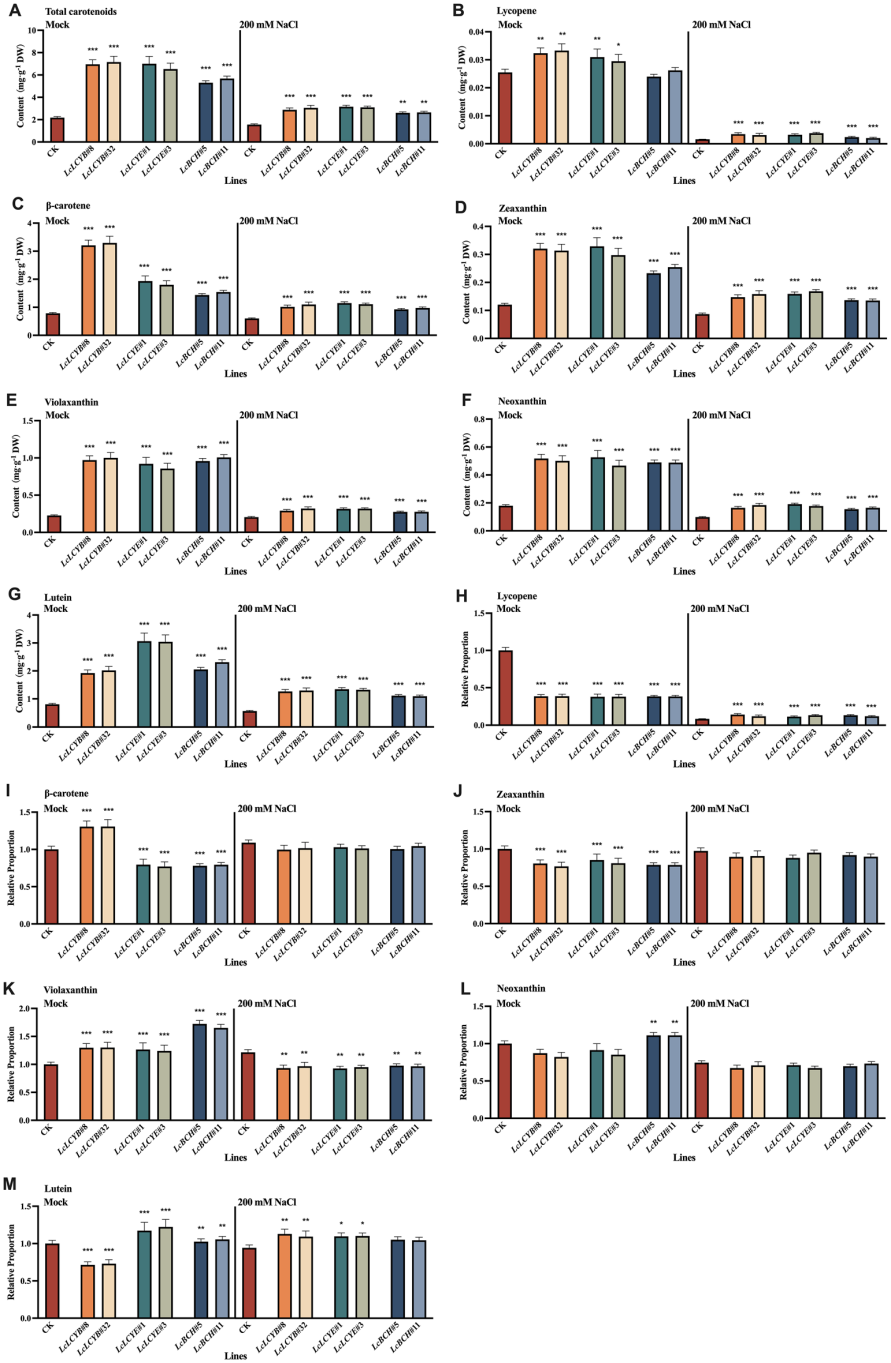
Effects of salt stress on carotenoid content in tobacco seedlings. Content of (**A**) total carotenoids, (**B**) lycopene, (**C**) β-carotene, (**D**) zeaxanthin, (**E**) violaxanthin, (**F**) neoxanthin and (**G**) lutein; relative proportion of (**H**) lycopene, (**I**) β-carotene, (**J**) zeaxanthin, (**K**) violaxanthin, (**L**) neoxanthin (**M**) lutein. Data were obtained from three independent experiments, and analysis of variance were performed. The results were presented as means ± standard deviation. *P* values of < 0.05 (*), < 0.01 (**) and < 0.001 (***) were considered to be significant statistically. 200 mM NaCl labelled samples were collected from the seedlings treated with 200 mM NaCl for 2 weeks while *Mock* labelled were collected from fresh water treated seedlings.

In order to further clarify the correlation between active oxygen scavenging and *LcLCYB*, *LcLCYE*, and *LcBCH* overexpression, changes in total carotenoid content (Fig. 5A), the content of main pigment including lycopene, β-carotene, zeaxanthin, violaxanthin, neoxanthin and lutein (Fig. 5B–G) and the proportion of each of them (Fig. 5H–M) were analyzed. Under 200 mM NaCl treatments, the total carotenoid content and each detected pigment content both decreased significantly, while the *35S:LcLCYB*, *35S:LcLCYE*, and *35S:LcBCH* tobaccos also maintained a higher level of carotenoid content compared with the CK plants. Furthermore, under NaCl treatments, a much higher consumption of the main carotenoid was detected in *35S:LcLCYB*, *35S:LcLCYE*, and *35S:LcBCH* tobacco seedlings in response to the oxidative stress, it showed that there was a good correlation between carotenoid content and salt tolerance (Fig. 5A–G).

In terms of the detected pigments, the proportion of lycopene decreased significantly greater, indicating an important role in response to the antioxidative stress (Fig. 5H). Meanwhile, the proportion of violaxanthin in *35S:LcLCYB*, *35S:LcLCYE*, and *35S:LcBCH* tobacco seedlings decreased significantly after NaCl treatments (Fig. 5K). These results demonstrated that the over-accumulation of violaxanthin, besides lycopene and the other pigments, might play crucial roles in response to the NaCl treatments in *35S:LcLCYB*, *35S:LcLCYE*, and *35S:LcBCH* tobacco seedlings.

### Genetic mechanism underlying overexpression of *LcLCYB*, *LcLCYE*, and *LcBCH* tobaccos improved tolerance upon the salt stress

**Figure 6:**
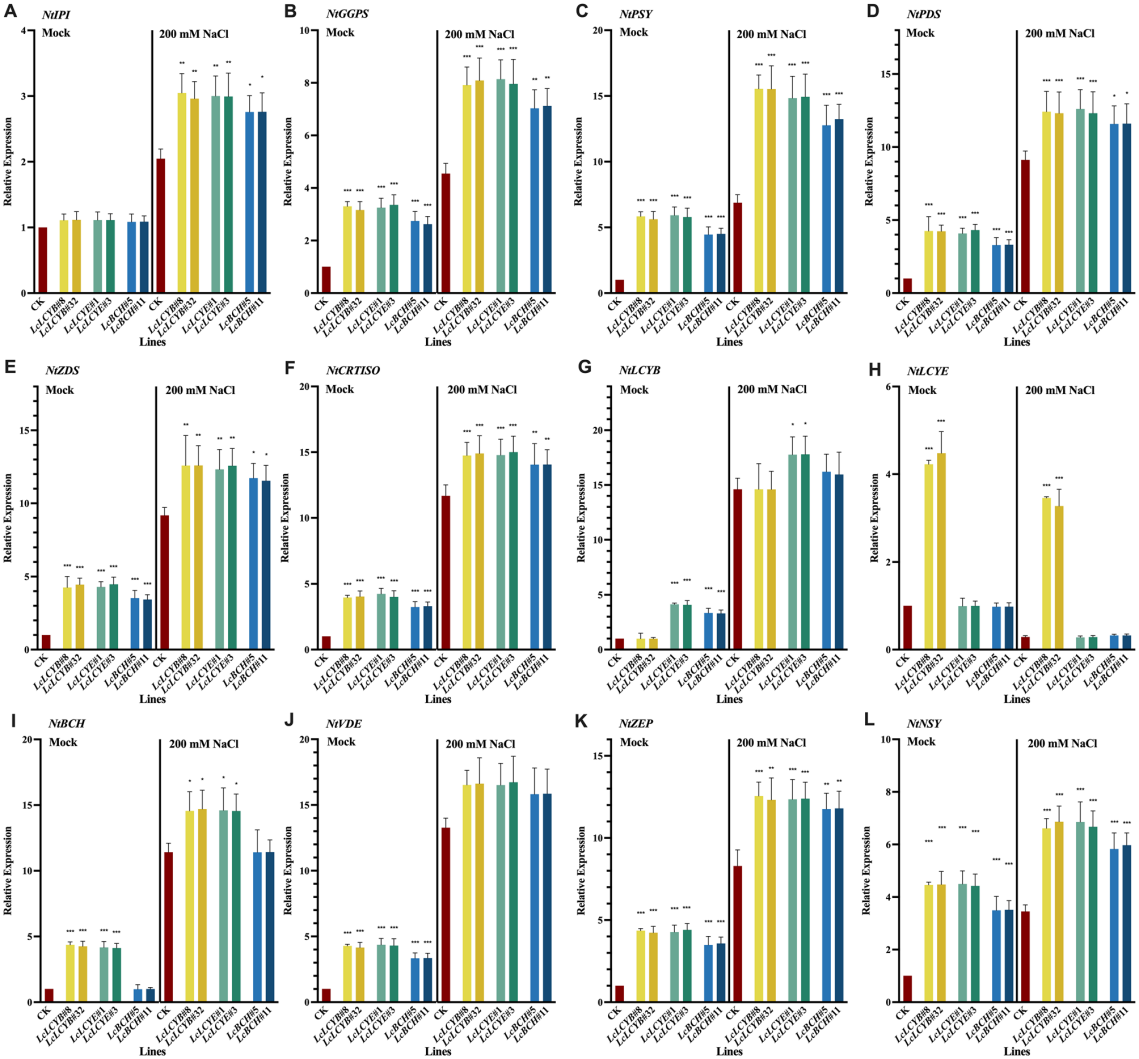
Effects of salt stress on endogenous carotenoid biosynthesis gene expression level in tobacco seedlings. Gene expression levels of (**A**) carotene isomerase (*NtIPI*), (**B**) geranylgeranyl pyrophosphate synthase (*NtGGPS*), (**C**) phytoene synthase (*NtPSY*), (**D**) phytoene desaturase (*NtPDS*), (**E**) ζ-carotene desaturase (*NtZDS*), (**F**) carotene isomerase (*NtCRTISO*), (**G**) lycopene β-cyclase (*NtLCYB*), (**H**) lycopene ε-cyclase (*NtLCYE*), (**I**) β-carotene hydroxylase (*NtBCH*), (**J**) violaxanthin de-epoxidase (*NtVDE*), (**K**) zeaxanthin epoxidase (*NtZEP*) and (**L**) neoxanthin synthase (*NtNSY*). *NtActin* was set as control. Data were obtained from three independent experiments, and analysis of variance were performed. The results were presented as means ± standard deviation. *P* values of < 0.05 (*), < 0.01 (**) and < 0.001 (***) were considered to be significant statistically. 200 mM NaCl labelled samples were collected from the seedlings treated with 200 mM NaCl for 2 weeks while *Mock* labelled were collected from fresh water treated seedlings.

To investigate the gene regulation network in the *35S:LcLCYB*, *35S:LcLCYE*, and *35S:LcBCH* overexpression tobaccos under NaCl treatments, the expression of the key carotenoid biosynthesis genes was analyzed in tobacco seedlings, including *NtIPI*, *NtGGPS*, *NtPSY*, *NtPDS*, *NtZDS*, *NtCRTISO*, *NtLCYB*, *NtLCYE*, *NtBCH*, and *NtVDE* (Fig. 6). Under normal conditions, the overexpression of *LcLCYB*, *LcLCYE*, and LcBCH significantly increased most carotenoid biosynthesis-related genes' expression level, except *NtIPI*, which gene is far away from these overexpression genes in the carotenoid biosynthesis pathway (Fig. 6A). Meanwhile, results showed that the overexpression of *LcLCYB*, *LcLCYE*, and *LcBCH* didn't affect the expression of the endogenous genes of *NtLCYB*, *NtLCYE*, and *NtBCH*, respectively (Fig. 6G–I).

After NaCl treatments, all the genes involved in the carotenoid biosynthesis pathway were up-regulated, and most genes were significantly higher in *35S:LcLCYB*, *35S:LcLCYE*, and *35S:LcBCH* tobaccos than in CK plants (Fig. 6). In addition, results showed that the significantly up-regulated genes were the upper-stream ones of the carotenoid biosynthesis pathway, in the *35S:LcLCYB*, *35S:LcLCYE*, and *35S:LcBCH* tobaccos, indicating that the overexpression of these genes promoted the carotenoid biosynthesis process, under both normal and NaCl treatment conditions (Fig. 6A–F). Genes, which were responsible for the carotenoid degradation, were also determined by qRT-PCR. The expression of *NtZEP* and *NtNSY* were significantly higher in *35S:LcLCYB*, *35S:LcLCYE*, and *35S:LcBCH* tobaccos than the CK plants both under normal and NaCl treatment conditions, indicating a higher speed of carotenoid consumption in response to the salt stress (Fig. 6K, L).

**Figure 7:**
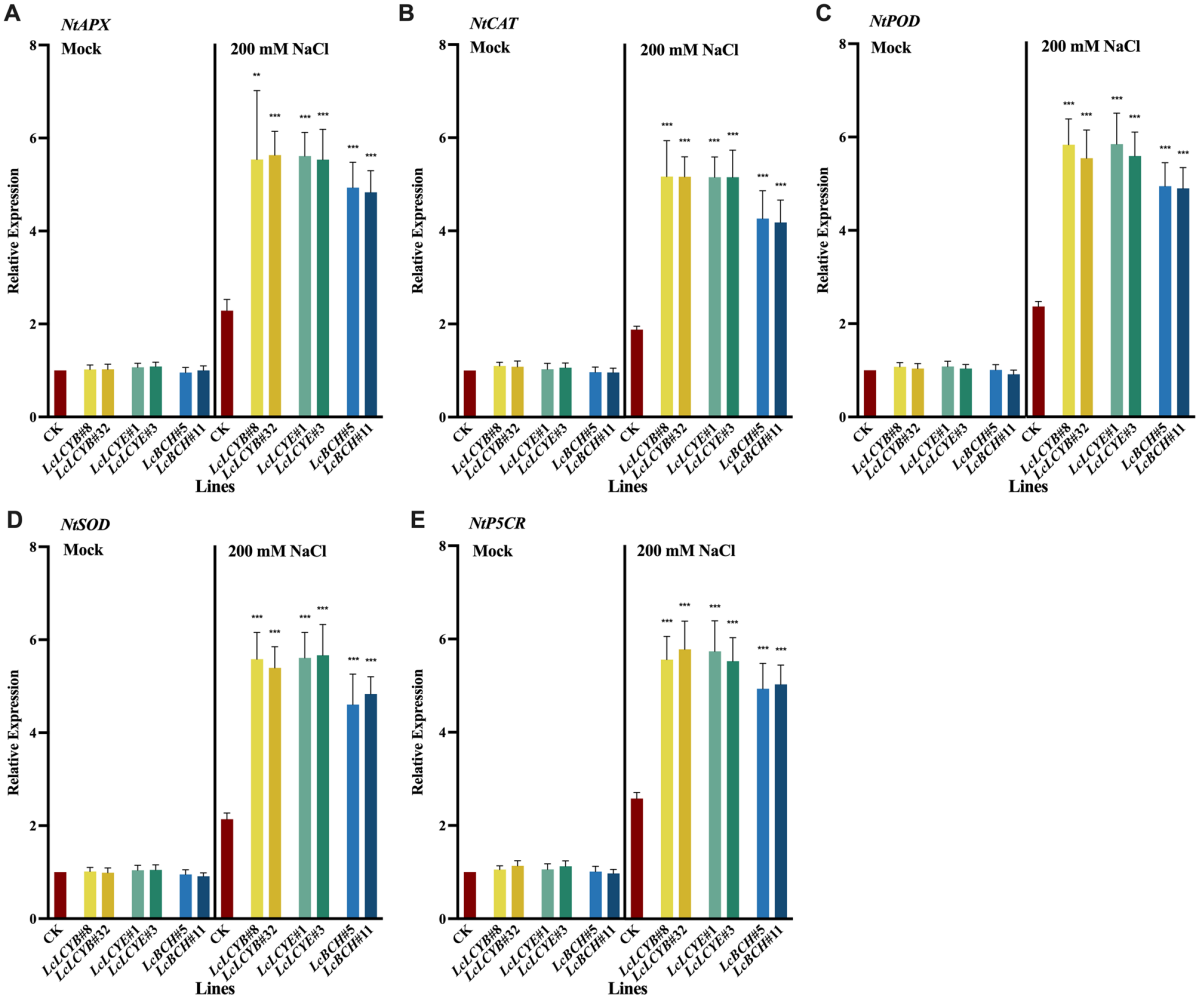
Effects of salt stress on stress-related gene expression level in tobacco seedlings. Expression levels of (**A**) ascorbate peroxidase (*NtAPX*), (**B**) catalase (*NtCAT*), (**C**) peroxidase (*NtPOD*), (**D**) superoxide dismutase (*NtSOD*) and (**E**) pyrroline-5-carboxylate reductase (*NtP5CR*). *NtActin* was set as control. Data were obtained from three independent experiments, and analysis of variance were performed. The results were presented as means ± standard deviation. *P* values of < 0.05 (*), < 0.01 (**) and < 0.001 (***) were considered to be significant statistically. 200 mM NaCl labelled samples were collected from the seedlings treated with 200 mM NaCl for 2 weeks while *Mock* labelled were collected from fresh water treated seedlings.

Coding genes of enzymes, which responsible for ROS scavenging, were analyzed, including ascorbate peroxidase (*NtAPX*), catalase (*NtCAT*), peroxidase (*NtPOD*), and superoxide dismutase (*NtSOD*), showing that these genes' expression level was significantly higher in *35S:LcLCYB*, *35S:LcLCYE*, and *35S:LcBCH* tobaccos than the CK plants under NaCl treatments (Fig. 7A–D). Expression of *NtP5CR* (pyrroline-5-carboxylate reductase), the key gene of the proline biosynthesis pathway, was also significantly higher in *35S:LcLCYB*, *35S:LcLCYE*, and *35S:LcBCH* tobaccos under NaCl treatments (Fig. 7E). All the results above suggested that the overexpression of *LcLCYB*, *LcLCYE*, and *LcBCH* may enhance tobacco seedlings' salt stress by accelerating the carotenoid biosynthesis, degradation, and ROS scavenging process.

### Accumulation of endogenous ABA in *LcLCYB*, *LcLCYE*, and *LcBCH* overexpression tobaccos also enhanced plants salt tolerance

**Figure 8:**
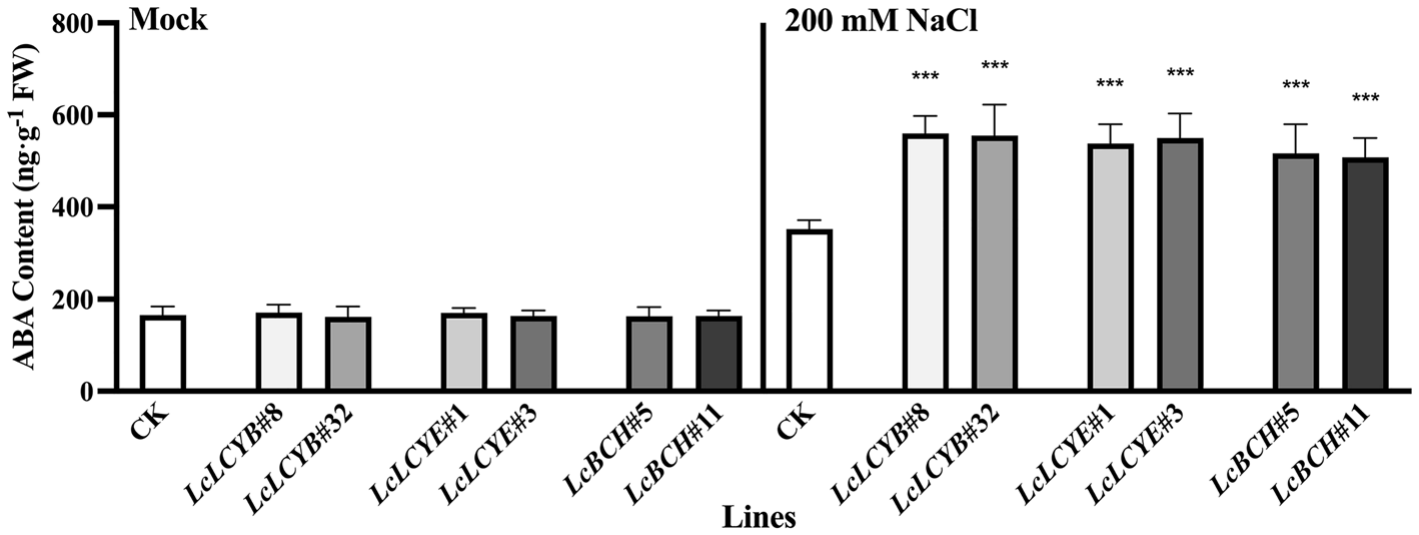
Determination of abscisic acid (ABA) content in tobacco seedlings. Data were obtained from three independent experiments, and analysis of variance were performed. The results were presented as means ± standard deviation. 200 mM NaCl labelled samples were collected from the seedlings treated with 200 mM NaCl for 2 weeks while *Mock* labelled were collected from fresh water treated seedlings.

To investigate the biological function of ABA, the endogenous ABA content in each overexpressor was analyzed. The results demonstrated that the ABA level increased in CK plants, as well as in *LcLCYB*, *LcLCYE*, and *LcBCH* overexpression tobaccos, when exposed to salt stress. Moreover, the accumulation of ABA was significantly higher in the overexpression lines compared to the CK plants. Under normal conditions, no significant difference in ABA content was observed between CK and overexpression lines (Fig. 8). The overexpression of carotenoid biosynthesis genes *LcLCYB*, *LcLCYE*, and *LcBCH* led to an increase in endogenous ABA accumulation, thereby enhancing the salt tolerance of tobaccos.

## Discussion

Carotenoids play essential roles in various metabolic processes, such as plant photosynthesis and hormone regulation. The underlying process of carotenoid biosynthesis has been studied in detail, but the mechanism of carotenoid biosynthesis regulations and its responses to environmental stress still remains unclear. As traditional Chinese herbal medicine, wolfberry (*L. chinense*) has strong tolerance to several abiotic stresses such as salt and drought and widely spreads in the semi-saline area of China^32^. The ripened red fruits of wolfberry contain various metabolites such as carotenoids, which show high anti-oxidative activity and nutritional values and are beneficial to humans and the plant itself^33^.

The carotenoid biosynthetic gene isolated from wolfberry is ideal for its functional and mechanism analysis. In our previous work, the overexpression of the three key genes involved in the desaturation process of carotenoid biosynthesis, *LcPDS*, *LcZDS*, and *LcCRTISO*, increased total carotenoid accumulation and salt tolerance of transgenic tobaccos. In this study, the key genes *LcLCYB*, *LcLCYE*, and *LcBCH*, which are responsible for the cyclization and hydroxylation, were isolated and analyzed in detail. Their overexpression significantly improved the total carotenoid content and salt tolerance of plants.

Tobacco overexpressing *LcLCYB*, *LcLCYE,* and, *LcBCH* significantly increased the content of each carotenoid component and the total carotenoid content, and this increase was positively correlated with the expression level of the exogenous gene (Figure S8 and S9). Meanwhile, the overexpression of the exogenous carotenoid biosynthesis genes boosted the accumulation of different carotenoid components to varying degrees. β-carotene, known as provitamin A, can be absorbed, digested, and converted to vitamin A^34^. Increasing the level of β-carotene is one of the most effective ways to optimize the nutritional structure of foods. Therefore, one of the goals of this study is to improve the biosynthesis and accumulation of β-carotene. The content of β-carotene increased in *LcLCYB* and *LcLCYE* by 3.15- and 1.39-folds, while the total carotenoid content increased by 2.24- and 2.11-fold, respectively (Fig. 1A, C).

In the carotenoid biosynthesis pathway, LCYB and LCYE both take lycopene as substrate and achieve the cyclization of a carotenoid skeleton. Their overexpression enhanced the accumulation of total carotenoids, slight content increase, and significant proportion decrease in lycopene in both overexpression tobaccos (Fig. 1A, B, H). Meanwhile, since LCYB catalyzes the formation of β-ringed carotenoids, its overexpression improved the accumulation of carotenoids containing β-rings in the plants, especially β-carotene (Fig. 1B, I). On the other hand, LCYE competes with LCYB for the same substrate. Its overexpression also promotes the consumption of lycopene and the formation of ε-ring carotenoids, showing an increased proportion of lutein (Fig. 1M). BCH catalyzes the synthesis of β-hydroxy-carotenoids from β-carotene. Overexpression of *LcBCH* increased the contents of total carotenoids, the β-cyclic carotenoids β-carotene, zeaxanthin, and violaxanthin, and decreased the β-carotene ratio (Fig. 1).

In summary, the proportion of the corresponding product of the overexpressed enzyme was higher, while the component of the enzyme–substrate decreased significantly in the carotenoid biosynthesis pathway, thus different expression patterns were observed in the three overexpression tobaccos (Fig. 1). Meanwhile, the overexpression of a branch-control enzyme significantly increased the accumulation and proportion of the corresponding branch production while decreasing the competitive branch ones (Fig. 1). Research indicates that LCYB coding genes overexpression may be the optimized selection for the enhancement of β-carotene content among three candidates, from a nutritional point of view, and the other ones provide supplementary choice for other purposes if needed.

When plants were suffering from NaCl treatments, the three *LcLCYB*, *LcLCYE*, and *LcBCH* overexpression tobaccos all showed enhanced salt tolerance. However, the underlying mechanism was not the same. Under NaCl treatments, the content of each carotenoid decreased while most endogenous carotenoid biosynthesis genes were significantly up-regulated (*NtLCYE* was exceptional) (Fig. 6). It can be inferred that the higher contents of carotenoids and endogenous carotenoid biosynthesis gene expression in the transgenic plants contributed to a higher salt resistance. Among the carotenoid components involved in the salt tolerance process, the decrease of each component was not even. Under NaCl treatments, lycopene was shown as a major component, with its content significantly reduced than the other carotenoids (Fig. 5B). As an essential node in carotenoid biosynthesis, lycopene functioned as a strong reductive component, tended to be consumed priorly to scavenge ROS, thus protecting membrane and other sensitive components from oxidization^17,35^.

Interestingly, the content of zeaxanthin decreased, but the proportion increased under NaCl treatments (Fig. 5D, J), while content and proportion of other components both decreased. (Fig. 5B, C, E–I, K–M). The most likely function of carotenoids was to be consumed as antioxidants under a programmed process to increase the tolerance when plants suffered from NaCl treatments, and the violaxanthin cycle was one of the proper explanations^36,37^. It was suggested that plants tend to accumulate more zeaxanthin by up-regulating the expression level of endogenous *VDE* and *ZEP* under NaCl treatments (Fig. 6J, K). The up-regulated *VDE* and *ZEP* accelerated the transformation of the components involved in the violaxanthin cycle, alleviating the oxidative stresses, thus enhancing plants' salt tolerance. And one of the plausible mechanisms may be that the overexpression of *LcLCYB* and *LcBCH* improved the synthesis and accumulation of violaxanthin and neoxanthin, which were naturally selected as protectors, thus achieving the enhancement.

As shown in the CK group, plants tend to down-regulate *NtLCYE* but up-regulate *NtLCYB* expression (Fig. 6G, H), thus driving more resources to the violaxanthin and neoxanthin synthesis, maybe also lead to increased ABA synthesis under salt stress^38,39^. The results indicated that a lower endogenous *LCYE* expression level might be preferred when plants exposure to abiotic stress. Comparing tobacco overexpressing wolfberry *PDS*, *ZDS*, *CRTISO*, *LCYB*, *LCYE*, and *BCH*, it was found that salt tolerance of transgenic plants was closely related to total carotenoid content. And the total carotenoid content maybe largely depends on the gene's catalytic function when the overexpression genes with a similar expression level^31^. More studies should be conducted to explore the decisive factor and underlying mechanism in the carotenoids biosynthesis pathway when plants suffered from salt stress.

Under salt stress, excessive ROS accumulation damages plant pigments, membrane systems, and enzyme systems, resulting in metabolic disorders and plant death^40^. The accumulation of ROS was assessed by detecting the levels of H_2_O_2_ and MDA, and found a lower level in the *LcLCYB*, *LcLCYE,* and *LcBCH* transgenic plants under normal and NaCl treatment conditions (Fig. 4D, E). It was indicated that extra accumulated carotenoids in overexpression plants could be used as reducing agents to eliminate ROS directly. Moreover, antioxidant enzymes were employed to detoxify the ROS and protect plants from oxidative stress. The enzyme activity and expression levels of *APX*, *CAT*, *POD,* and *SOD* were significantly increased in overexpression plants (Fig. 4G–I). The results show that the accumulated carotenoids could detoxify ROS by positively regulating the expression of antioxidant enzyme coding genes and increasing the activity of antioxidant enzymes.

Proline is one of the important antioxidative components in plants. It is synthesized and accumulated to eliminate reactive oxygen and quench singlet oxygen when oxidative stress occurs in plants. Under high osmotic conditions, proline protects plants from osmotic stress and increases plant salt tolerance^41^. Under NaCl treatments, expression level of the *P5CR*, rate-limiting enzyme gene in proline biosynthesis, was significantly increased in *35S:LcLCYB*, *35S:LcLCYE*, and *35S:LcBCH* transgenic tobaccos, and proline biosynthesis accelerated (Fig. 7E). Compared with the CK plants, much more accumulated proline, indicating that total carotenoid accumulation increases the ability of plants to utilize more proline to improve salt tolerance (Fig. 4F). In summary, carotenoids can be directly used as reducing agents and indirectly promote plant stress tolerance by promoting the activity of the antioxidant enzymes and *P5CR* expression.

Under normal circumstances, the production and elimination of ROS are in a steady-state during plant growth. When the ROS scavenging system is accelerated, the ROS content decreases, and the photosynthesis increases, as the accumulated ROS destroys the pigment and membrane system in the chloroplast^42^.

The chlorophyll content and photosynthesis (Pn and Fv/Fm) were significantly higher in the *LcLCYB*, *LcLCYE,* and *LcBCH* overexpressing plants than the CK plants (Fig. 4). Under normal conditions, the chlorophyll content increased, and the photosynthetic parameters (Pn and Fv/Fm) were significantly higher in the *LcLCYB*, *LcLCYE,* and *LcBCH* overexpressing plants (Fig. 4). The photosynthesis system was severely damaged under NaCl treatments, showing a lower chlorophyll content and decreased photosynthetic rate; meanwhile, the three overexpressors performed better on the photosynthesis than the CK plants (Fig. 4).

Tobacco plants overexpressing *LcLCYB*, *LcLCYE*, and *LcBCH* had higher cumulative antioxidant levels, more efficient antioxidant enzyme catalytic activity and more efficient ROS scavenging system, resulting in less ROS accumulation in leaves. Thus, the components involved in photosynthesis were also less damaged by ROS, showing higher chlorophyll content and better photosynthetic performance. It was indicated that the accumulation of carotenoids improved the antioxidant capacity of *LcLCYB*, *LcLCYE,* and *LcBCH* transgenic plants, promoted their photosynthesis, and meanwhile enhanced salt tolerance of the plants.

Increased expression of endogenous carotenoid biosynthesis genes except for its homologous gene in the three overexpression tobaccos (Fig. 6). This indicates that plants may accelerate the carotenoid biosynthesis process by actively regulating the expression level of endogenous carotenoid biosynthesis genes and regenerating new homeostasis to cope with the steady-state shock caused by overexpression of exogenous genes. It was also observed that when plants exposed to NaCl treatments, the expression of *LCYE* in the CK and *35S:LcBCH* plants was significantly reduced. However, the expression of *LCYE* was increased significantly in *35S:LcLCYB* under normal and NaCl treatments (Fig. 6H). Results indicated that there might be a relatively stable relationship between the β-branch and the ε-branch pathway, as the ε-branch product accumulation was much higher in the *35S:LcLCYB* plants. When *LCYB* expression increases significantly, *LCYE* expression increases, and vice versa. Similar phenomena were also found in previous work, and the specific regulatory mechanism was still unclear^43,44^. It is concluded that the relationship between β and ε-branch pathway is very important. Therefore, under normal and NaCl treatment conditions, plants tend to maintain this relationship by up-regulating *LCYE* in *35S:LcLCYB* plants.

As phytohormone, ABA plays critical roles in mediating a wide range of physiological activities when plants exposed to various abiotic stresses, particularly salt stress^45^. Carotenoids, which are precursors of ABA, accumulated in plants that overexpressing *LcLCYB*, *LcLCYE*, and *LcBCH* genes. When plants were subjected to salt stress, the excess carotenoids were rapidly converted to ABA, which contributed to the plants' ability to tolerate abiotic stresses in the overexpression plants. Additionally, the overexpression plants exhibited an efficient antioxidant system, characterized by antioxidant enzymes and the accumulation of antioxidant components. This system effectively detoxified ROS, reduces oxidative damage, and enhanced the plants' tolerance to salt stress.

## Methods

### Plant material growth conditions and NaCl treatments

The Chinese wolfberry (*L. chinense* Miller) and Tobacco (*N. tabacum* NC89) seeds used in this study were previously preserved in our laboratory. Plants used in this study were grown in a greenhouse of the College of Life Sciences, Dezhou University (Dezhou, Shandong, China), all operations were in accordance with relevant institutions, national and international norms and legislation in the study. The red fruits of wolfberry were harvested immediately once completely ripened, showing fully red and slightly dehydrated, about 7 weeks after pollination, and frozen with liquid nitrogen (Figure S1). Tobacco seeds were used in this study. After 3-d vernalization at 4 °C, the surface-sterilized tobacco seeds were sown in sterilized Petri dishes, which were supplemented with half-strength Murashige and Skoog (MS) basal salts, 1.0% (w/v) sucrose, pH 5.70, and 0.75% (w/v) agar powder.

For the NaCl treatments of tobacco seedlings, 5-d old seedlings were transferred to the 1/2 MS containing 200 mM NaCl for 14 days. Meanwhile, 14-d old seedlings sown in the soil were watered with 200 mM NaCl every second day in 2 weeks. The primary root length of seedlings was measured with a vernier caliper.

### Sequence characterization, cloning, and tobacco plants transformation

*LcLCYB* (GenBank Accession No. KP262047.1), *LcLCYE* (GenBank Accession No. KF768738.2), and *LcBCH* (GenBank Accession No. KF430643.1) were isolated from the cDNA of the ripened wolfberry fruits. Physicochemical characterization of proteins, including lengths of protein sequences, molecular weights (Da), and isoelectric point (pI), were analyzed online by the ExPASy bioinformatics resource portal (https://web.expasy.org/protparam/)^46^. The putative subcellular localization was predicted with CELLO v.2.5: subcellular localization predictor (http://cello.life.nctu.edu.tw/)^47,48^. Phylogenetic trees of each gene between referenced species were constructed by MEGA X^49^. Similarity and identity percentage of proteins were analyzed by Clustal Omega (https://www.ebi.ac.uk/Tools/msa/clustalo/) through multiple sequence alignments^50^. PFAM (http://pfam.xfam.org/) and SWISS-MODEL (https://swissmodel.expasy.org/) were used online to analyze the protein domain and tertiary structures^51–53^. The genetic and species information in the analysis was listed in Table S2.

Plasmids of pET-28a-*LcLCYB*, pET-28a-*LcLCYE*, and pET-28a-*LcBCH* were constructed for catalytic activity analysis in vitro. pET-28a-*LcLCYB* and pET-28a-*LcLCYE* were co-transformed with pACCRT-*EBI*, which carries genes coding geranylgeranyl diphosphate synthase (*crtE*), 15-cis-phytoene synthase (*crtB*), and phytoene desaturase (*crtI*). Meanwhile, plasmid pACCRT-*EBI*, pET-28a-*LcLCYB* was co-transformed with pET-28a-*LcBCH* into *E*. *coli* for the enzymatic activity analysis of *LcBCH*.

Genes were cloned into the binary vector pCambia2300 to generate *35S:LcLCYB*, *35S:LcLCYE*, and *35S:LcBCH* constructs, which were introduced into *Agrobacterium tumefaciens* C58 via electric shock (25 μF, 2000 V) and subsequently transformed into the tobacco plants through the leaf disc method, respectively^54^. Functional identification was accomplished in the homozygous transgenic tobaccos (T3) compared with the control plants (CK), which contain an empty vector (*35S*), and confirmed in the T4 generation homozygous seedlings. Primers used for vector construction were listed in Table S3.

### Carotenoid extraction and HPLC analysis

The liquid nitrogen frozen samples were vacuum-dried with the freeze drier, then grounded to a fine powder. A 0.1 g dried sample was mixed with 10 mL of potassium hydroxide/methanol (6:100, w/v) in a 50 mL tube and incubated at 60 °C for 20 min. The sample was cooled to room temperature, 20 mL of ether was added and mixed well. After partitioning for 30 min, the upper layer was collected and dried with nitrogen gas. HPLC condition was set as follows: the dried sample obtained from the above procedure was dissolved in the acetone to 50 μL and separated with the mobile phase of isopropanol/methanol/acetonitrile (5:10:85, v/v/v) on a nucleosil 100-3 C18, 250 × 4.6 mM (MN, Germany) column; injection volume: 20 μL; column temperature: 32 °C; flow rate: 1 mL/min; detector: Kontron DAD 440; wavelength detected: 450 nm. For the identification and quantification of each carotenoid, the standard reference compound was also loaded and detected the same as above (Figure S10 and Table S4). To protect the samples from decomposition, all the apparatuses used in extraction and chromatography analysis were wrapped with foil, and solvents involved were saturated with nitrogen gas.

### RNA isolation and quantitative RT-PCR analysis

Total RNA was extracted from approximately 0.1 g leaves with RNeasy plant mini kit (QIAGEN). Synthesis of the first strand of cDNA was performed using 2 μg of total RNA primed with oligo (dT)_18_ primers by PrimeScript™ RT Master Mix (for Real-time) kit. Real-time PCR was performed with a model real-time PCR system (Agilent Mx3000P) using SYBR Premix Ex Taq TM (Takara) according to the manufacturer's guidance. To normalize the results, *NtActin* was amplified for each sample as a control to calculate ΔCt (ΔCt = Control Ct − Target Ct) values, and gene-specific primers involved in real-time PCR were listed in Table S5. Thermal cycling conditions included an initial incubation at 95 °C for 30 s, followed by 40 cycles of 95 °C for 15 s, 58 °C for 35 s, and 72 °C for 15 s. All the samples were run in triplicate, and the standard deviation was calculated.

### Phenotypic and physiological analysis

Seed germination rate was defined as the number of seeds obvious emergence of the radicle through the seed coat per 100 seeds. Relative water content was detected following the methods by Barrs^55^. Photosynthetic parameter analysis was applied according to the manufacture's illustration. LI 6400 portable apparatus (LI-COR, USA) with a pulse amplitude modulation (PAM-2000) portable fluorometer was employed to determine the photosynthesis rate (Pn) and photochemical efficiency of photosystem II (Fv/Fm). The experiments were performed at 11:30–14:00 on sunny days, and dark-adaptation of the plants was applied for 30 min by using foil-covered boxes.

0.1 g fresh leaf sample was quickly frozen with liquid nitrogen and dried with a freeze drier. Freeze-dried leaf sample was employed in each analysis of chlorophyll, H_2_O_2_, and MDA content. Deionized water treated with the same procedures was set as mock. Chlorophyll in the leaves was extracted with anhydrous ethanol and quantified spectrophotometrically at 645 nm and 663 nm as the description by Wintermans and De Mots^56^. Zhang's procedure was used to determine H_2_O_2_^57^. Pre-cold acetone was applied in the extraction of H_2_O_2_, 5% TiSO_4_ solution and 25% ammonia solution was used in the transformation and precipitation of H_2_O_2_, concentrated H_2_SO_4_ was used in the re-dissolution of titanium-peroxide complex, and the absorbance of the complex at 415 nm was recorded. For the estimation of MDA, the protocol improved by Hodges was followed^58^. MDA was extracted with 10% trichloroacetic acid solution and then co-heated with 0.6% thiobarbituric acid solution with boiling water bath. The absorbance of the cooled sample was measured at 450 nm, 532 nm and 600 nm. The assessment of proline content was performed by a procedure given by the Bates et al.^59^. 3% sulphosalicylic acid solution was employed in the extraction of proline with a boiling water bath. Sample solution was cooled, filtered and mixed with acetic acid and ninhydrin solution. Toluene was mixed with the colorized sample and then partitioned for 10 min. The supernatant was collected and measured with a spectrometer at 520 nm.

0.1 g fresh leaf sample was powdered with frozen-nitrogen and suspended with ice-cold potassium phosphate buffer (100 mM, pH 7.0) and PVP (1%) for the evaluation of antioxidant enzyme activities, including CAT (EC 1.11.1.6), POD (EC 1.11.1.7), and SOD (EC 1.15.1.1), based on the methods by Donahue et al.^60^. Samples and following buffers were stored in ice-bath. Blank buffer without leaf sample treated with sample procedure was set as mock. CAT activity was spectrophotometrically determined by measuring the decomposition of H_2_O_2_. 2 mL 60 mM H_2_O_2_ solution was added to 2 mL sample solution. The absorbance at 240 nm was employed for the determination after 5 min reaction in a 25 °C incubator. POD activity was determined by measuring the increase of absorbance at 420 nm, which caused by the reaction of 4-methylcatechol with H_2_O_2_. 2 mL reaction buffer containing 10 mM 4-methylcatechol and 10 mM H_2_O_2_ was added to 2 mL sample solution. Reaction was applied in a 25 °C incubator for 5 min. An absorbance measurement at 420 nm was collected for the calculation. SOD activity was determined by measuring the inhibition in the photochemical reduction of nitroblue tetrazolium. 2 mL reaction buffer containing 0.2 mM EDTA, 26 mM methionine, 150 µM nitroblue tetrazolium and 4 µM riboflavin was added into 2 mL sample solution. Once the sample solution and reaction buffer mixed well, the mixture was placed under two 15-Watt fluorescent lamps in a 25 °C incubator for initiation of the reaction. The reaction was terminated after 10 min by removing the light source. The reaction product was measured at 560 nm spectrophotometrically.

### Extraction and quantification of endogenous ABA

The endogenous ABA in tobacco leaves were extracted based on the methods described by Sano and Marion-Poll^45^ and then quantified with ELISA Kit (EY-01H3384, Yi Yan, Shanghai, China) in accordance with the manufacturer's instructions. 0.1 g fresh leaf sample was collected and powdered with liquid nitrogen. Fine powdered sample was then mixed with 1 mL 80% methanol vigorously, and then centrifuge the sample at 12,000*g* for 20 min. the supernatant was collected and dried with nitrogen gas flow. Re-dissolve the sample with potassium phosphate buffer (100 mM, pH 7.0), mixed vigorously, and then centrifuge the sample at 12,000*g* for 20 min. 50 µL of samples was loaded into 96-well plate offered by kit and co-incubated with 100 µL HRP (horseradish peroxidase)-labeled antibody solution for 60 min at 37 °C. The sample solution was then removed. Wash the wells with washing buffer 5 times and dry with air flow with 5 min. 50 µL substrate A solution and 50 µL substate B solution was added into the well and incubated for 15 min at 37 °C. Add 50 µL termination solution to each well and measure the absorbance at 450 nm.

### Statistical analysis

All data were collected at least three independent replicates, and presented as means ± standard deviation. Prism 7 software (GraphPad Software Inc, USA) via one-way analysis of variance was accomplished, with *P* values of < 0.05 (*), < 0.01 (**) and < 0.001 (***) were considered to be statistically significantly different from each other.

### Supplementary Information

Supplementary Information.

## Data Availability

All data generated or analyzed during this study are included in this published article and its supplementary information files.

## Abbreviations

ABA: Abscisic acid
APX: Ascorbate peroxidase
BCH: β-Carotene hydroxylase
CAT: Catalase
CRTISO: Carotene isomerase
DMAPP: Dimethylallyl diphosphate
DW: Dry weight
FAD: Flavin adenine dinucleotide
GGPS: Geranylgeranyl pyrophosphate synthase
HRP: Horseradish peroxidase
HPLC: High performance liquid chromatography
IPI: Isopentenyl pyrophosphate isomerase
IPP: Isopentenyl diphosphate
LCYB: Lycopene β-cyclase
LCYE: Lycopene ε-cyclase
MDA: Malondialdehyde
MS: Murashige and Skoog
P5CR: Pyrroline-5-carboxylate reductase
PDS: Phytoene desaturase
POD: Peroxidase
PS: Photosynthesis system
PSY: Phytoene synthase
ROS: Reactive oxygen species
SOD: Superoxide dismutase
VDE: Violaxanthin de-epoxidase
ZDS: ζ-Carotene desaturase
ZEP: Zeaxanthin epoxidase
