# Supplementary material for: Carotenoid biosynthesis genes LcLCYB, LcLCYE, and LcBCH from wolfberry confer increased carotenoid content and improved salt tolerance in tobacco
Source: Sci Rep. 2024 May 8;14:10586. doi: 10.1038/s41598-024-60848-3 (PMC11079049; doi:10.1038/s41598-024-60848-3)
Supplement: Supplementary file 1 — Supplementary Information. [file 41598_2024_60848_MOESM1_ESM.pdf]

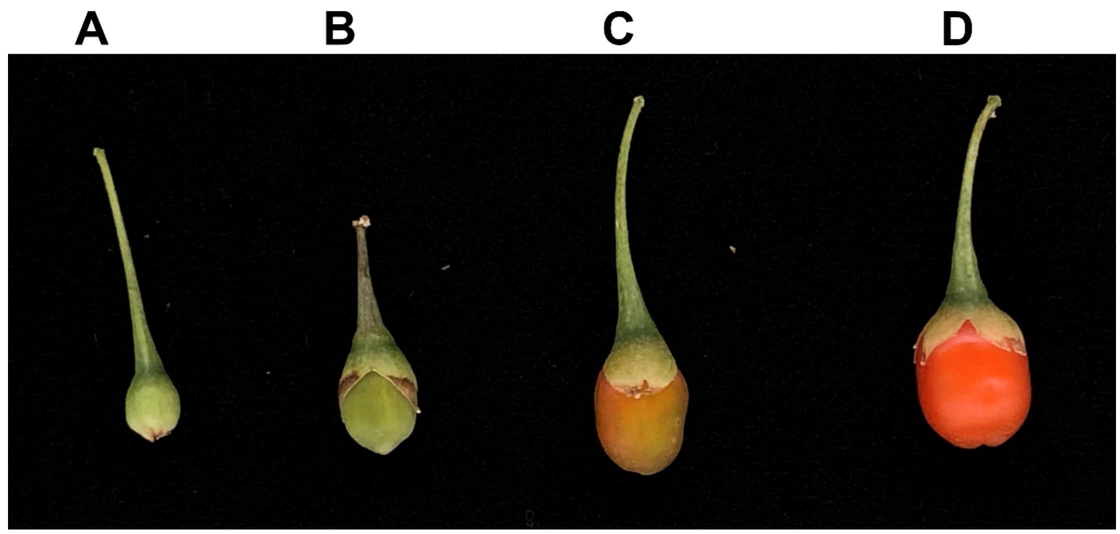

Supplementary Figure 1. Different ripening stages of wolfberry fruits. The genes were isolated from the stage (D), the red ripened fruits.

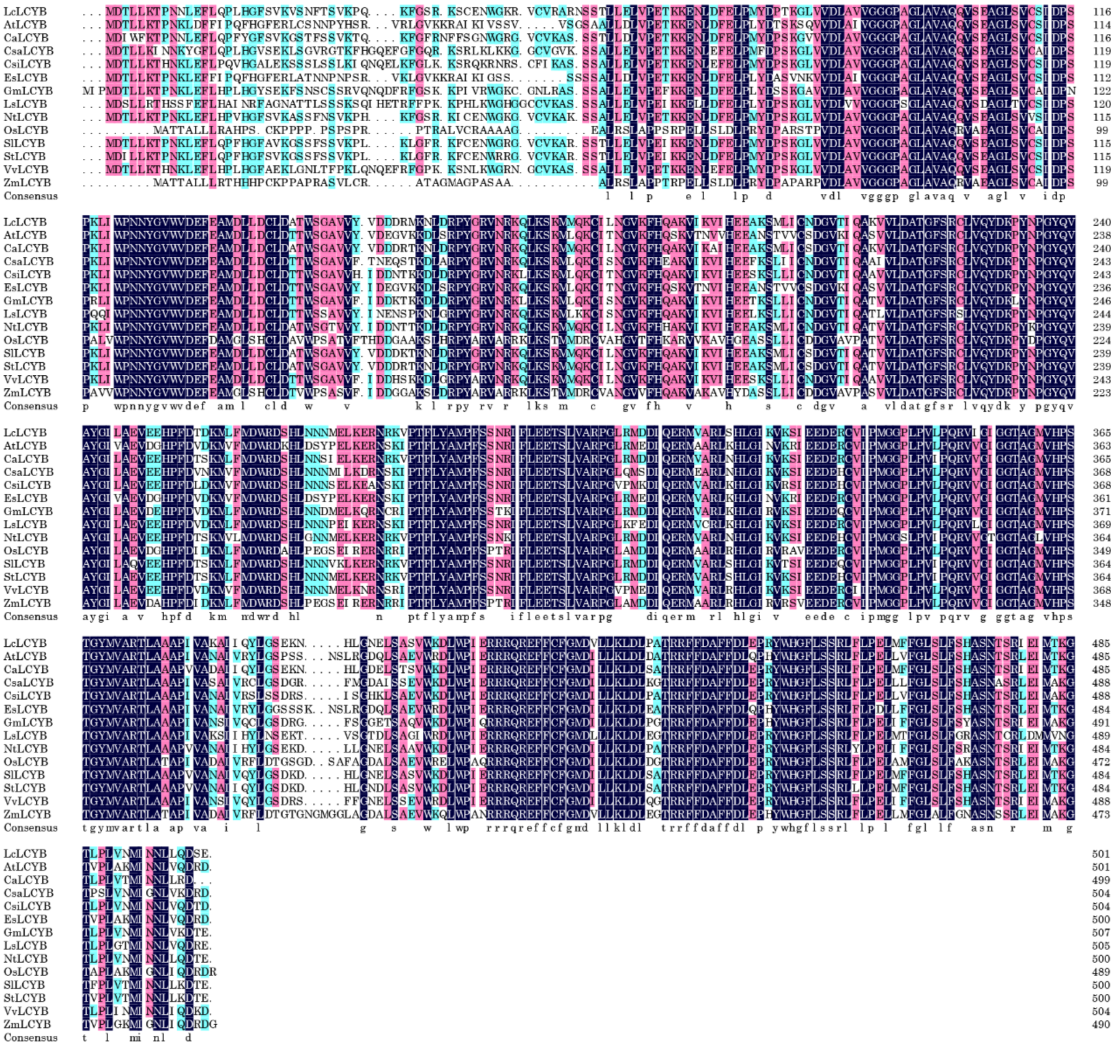

Supplementary Figure 2. Multiple sequence alignments of LcLCYB with the selected corresponding proteins. AtLCYB (*Arabidopsis thaliana*, NP_187634), CaLCYB (*Capsicum annuum*, PHT71462), CsaLCYB (*Cucumis sativus*, XP_004150761), CsiLCYB (*Citrus sinensis*, AAM21152), EsLCYB (*Eutrema salsugineum*, XP_006407592), GmLCYB (*Glycine max*, XP_003554132), LsLCYB (*Lactuca sativa*, XP_023755637), NtLCYB (*Nicotiana tabacum*, NP_001311716), OsLCYB (*Oryza sativa*, XP_015627234), SlLCYB (*Solanum lycopersicum*, XP_010312096), StLCYB (*Solanum tuberosum*, XP_006351264), VvLCYB (*Vitis vinifera*, XP_002275769) and ZmLCYB (*Zea mays*, NP_001169155).

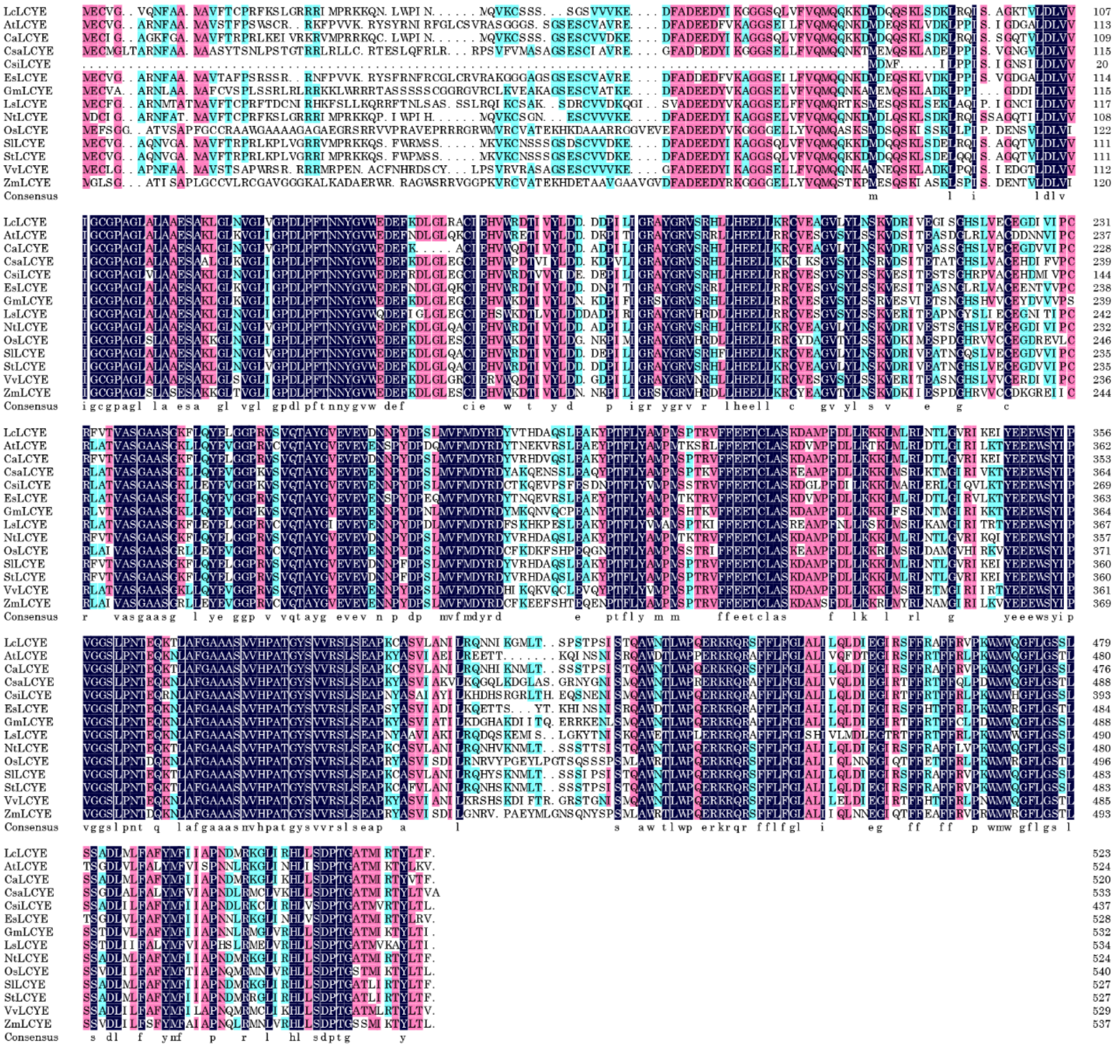

Supplementary Figure 3. Multiple sequence alignments of LcLCYE with the selected corresponding proteins. AtLCYE (*Arabidopsis thaliana*, NP_200513), CaLCYE (*Capsicum annuum*, PHT73568), CsaLCYE (*Cucumis sativus*, XP_004141172), CsiLCYE (*Citrus sinensis*, AAS48096), EsLCYE (*Eutrema salsugineum*, XP_006401252), GmLCYE (*Glycine max*, XP_003533775), LsLCYE (*Lactuca sativa*, XP_023756930), NtLCYE (*Nicotiana tabacum*, XP_016467034), OsLCYE (*Oryza sativa*, XP_015622198), SlLCYE (*Solanum lycopersicum*, NP_001234337), StLCYE (*Solanum tuberosum*, XP_006353544), VvLCYE (*Vitis vinifera*, RVW43956) and ZmLCYE (*Zea mays*, AQK96213).

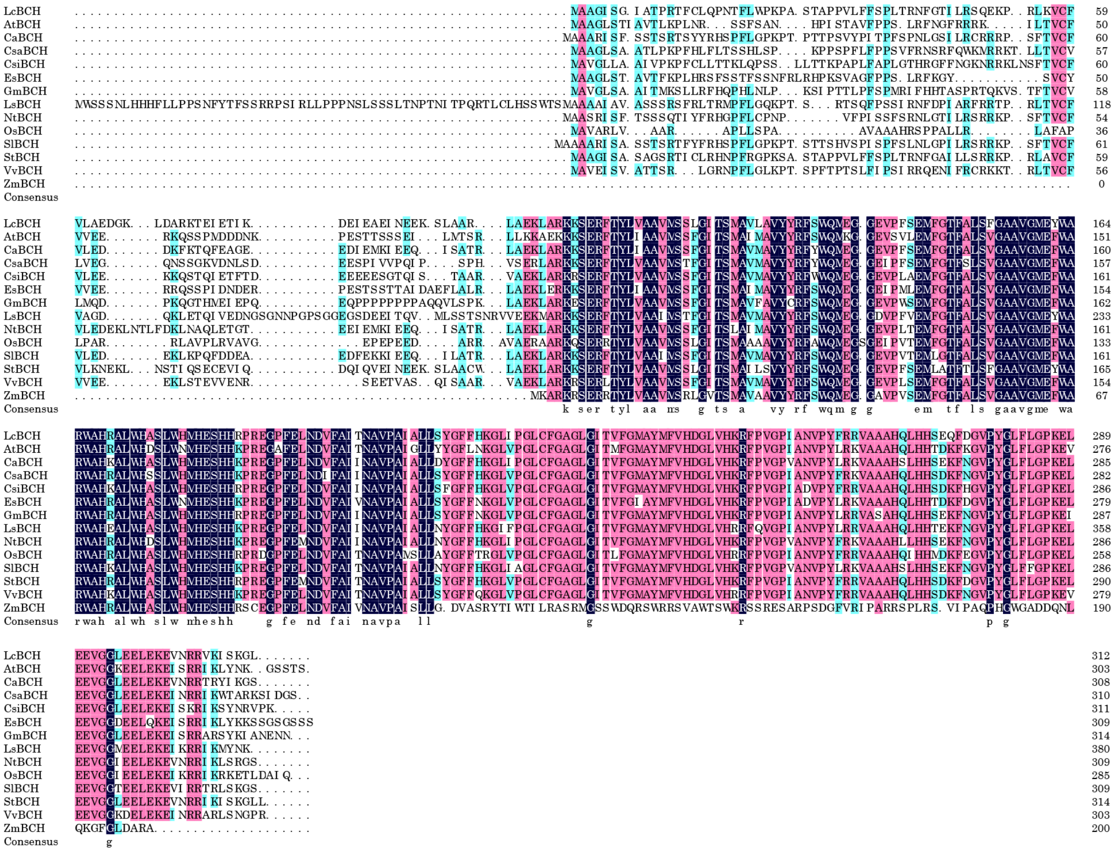

Supplementary Figure 4. Multiple sequence alignments of LcBCH with the selected corresponding proteins. AtBCH (*Arabidopsis thaliana*, NP_200070), CaBCH (*Capsicum annuum*, KAF3668875), CsaBCH (*Cucumis sativus*, XP_004140758), CsiBCH (*Citrus sinensis*, NP_001275830), EsBCH (*Eutrema salsugineum*, XP_006413280), GmBCH (*Glycine max*, NP_001242409), LsBCH (*Lactuca sativa*, XP_023769464), NtBCH (*Nicotiana tabacum*, NP_001313021), OsBCH (*Oryza sativa*, XP_015628773), SlBCH (*Solanum lycopersicum*, NP_001234348), StBCH (*Solanum tuberosum*, ADF28628), VvBCH (*Vitis vinifera*, RVW41947) and ZmBCH (*Zea mays*, NP_001105865).

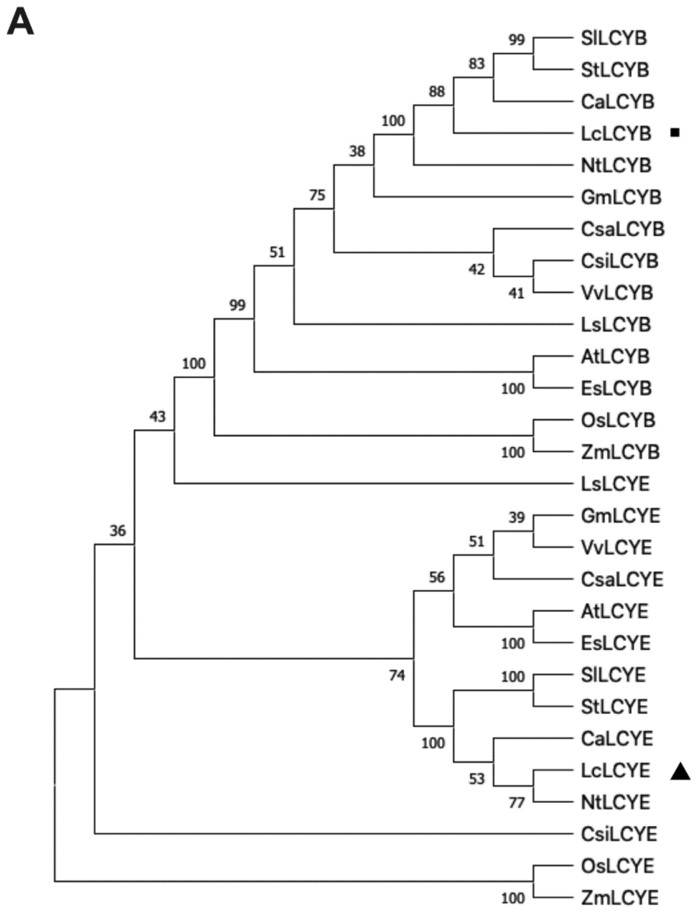

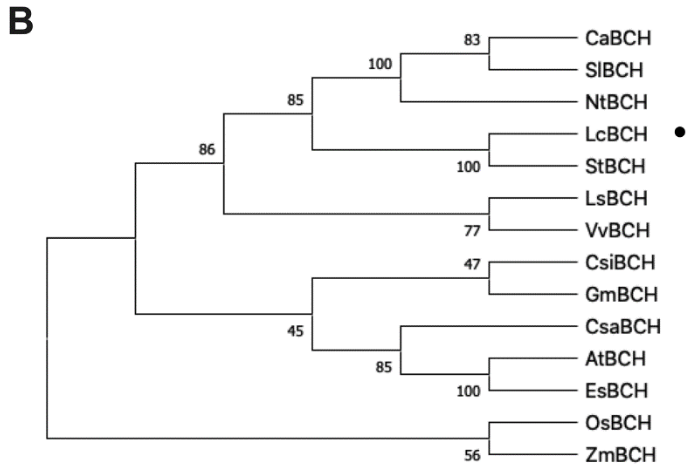

Supplementary Figure 5. Phylogenic analysis of **(A)** LCYB and LCYE and **(B)** BCH were constructed using the Neighbor-Joining method with MEGA X software. The LcLCYB, LcLCYE, and LcBCH were indicated with ■, ▲ and ●, respectively.

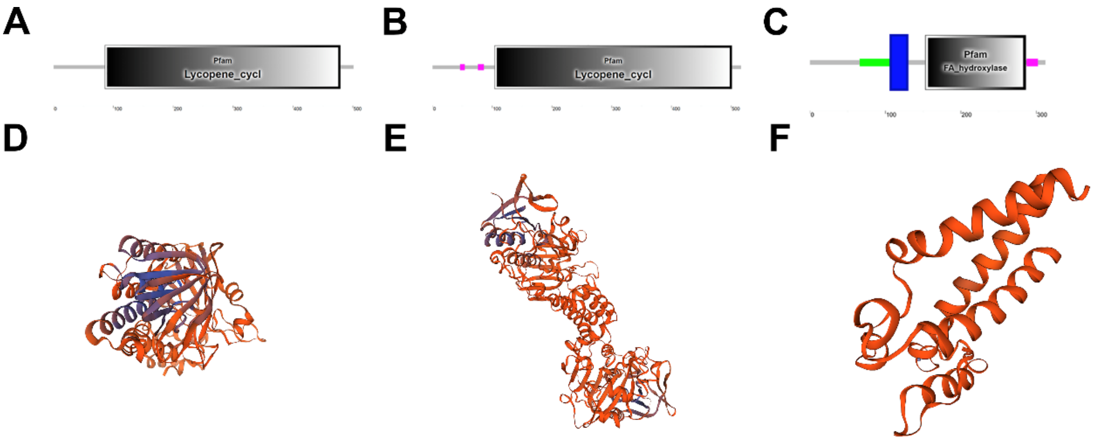

Supplementary Figure 6. Conserved domain analysis of **(A)** LcLCYB, **(B)** LcLCYE, and **(C)** LcBCH were accomplished with PFAM online tools (http://pfam.xfam.org/), and 3D structure prediction of **(D)** LcLCYB, **(E)** LcLCYE, and **(F)** LcBCH were applied with SWISS-MODEL Workspace (https://swissmodel.expasy.org/).

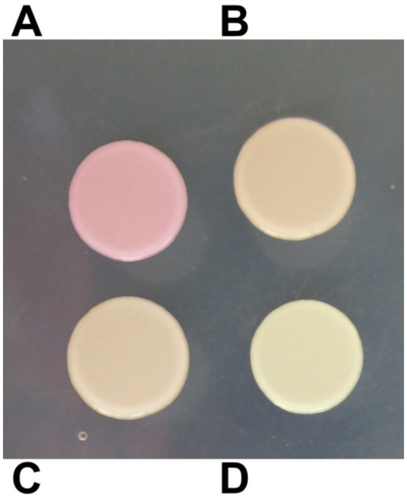

Supplementary Figure 7. The color complementation of *LcLCYB*, *LcLCYE,* and *LcBCH* in engineered *E.coli*. Recombined *E.coli* strains containing plasmid **(A)** pACCRT-*EBI* (carring genes coding geranylgeranyl diphosphate synthase, 15-cis-phytoene synthase, and phytoene desaturase), **(B)** pACCRT-*EBI* and pET-28a-*LcLCYB*, **(C)** pACCRT-*EBI* and pET-28a-*LcLCYE*, and **(D)** pACCRT-*EBI*, pET-28a-*LcLCYB* and pET-28a-*LcBCH* were displayed.

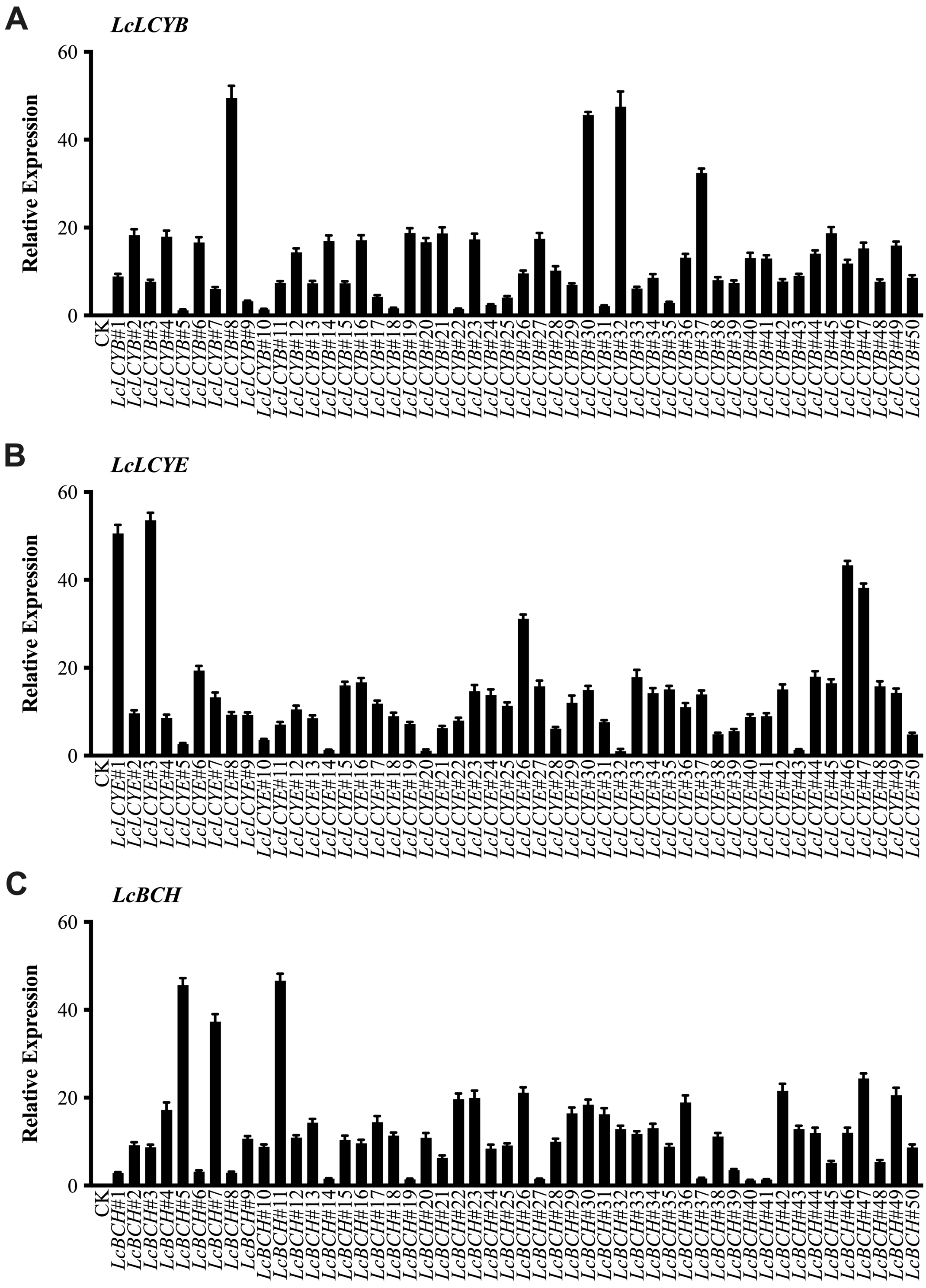

Supplementary Figure 8. Expression levels of **(A)** *LcLCYB*, **(B)** *LcLCYE*, and **(C)** *LcBCH* in transgenic tobaccos were measured by Real-time PCR, and results were normalized to reference gene *NtActin*. Tobacco overexpression lines were named as *Gene#Number*. Data were obtained from three independent experiments and performed as means ± standard deviation.

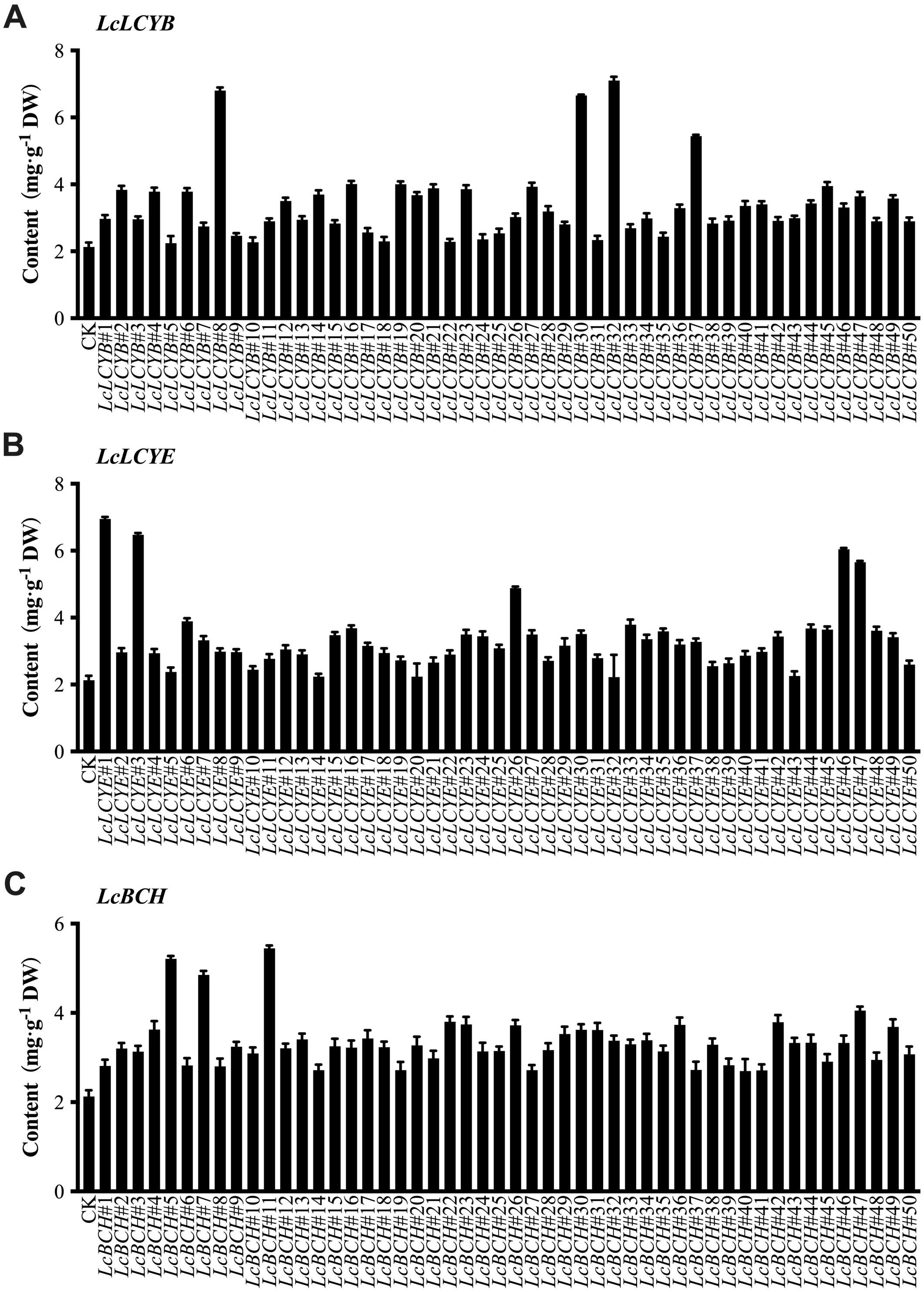

Supplementary Figure 9. Total carotenoid contents in **(A)** *LcLCYB*, **(B)** *LcLCYE*, and **(C)** *LcBCH* in transgenic tobacco leaves. Tobacco overexpression lines were named as *Gene#Number*. Data were obtained from three independent experiments and performed as means ± standard deviation.

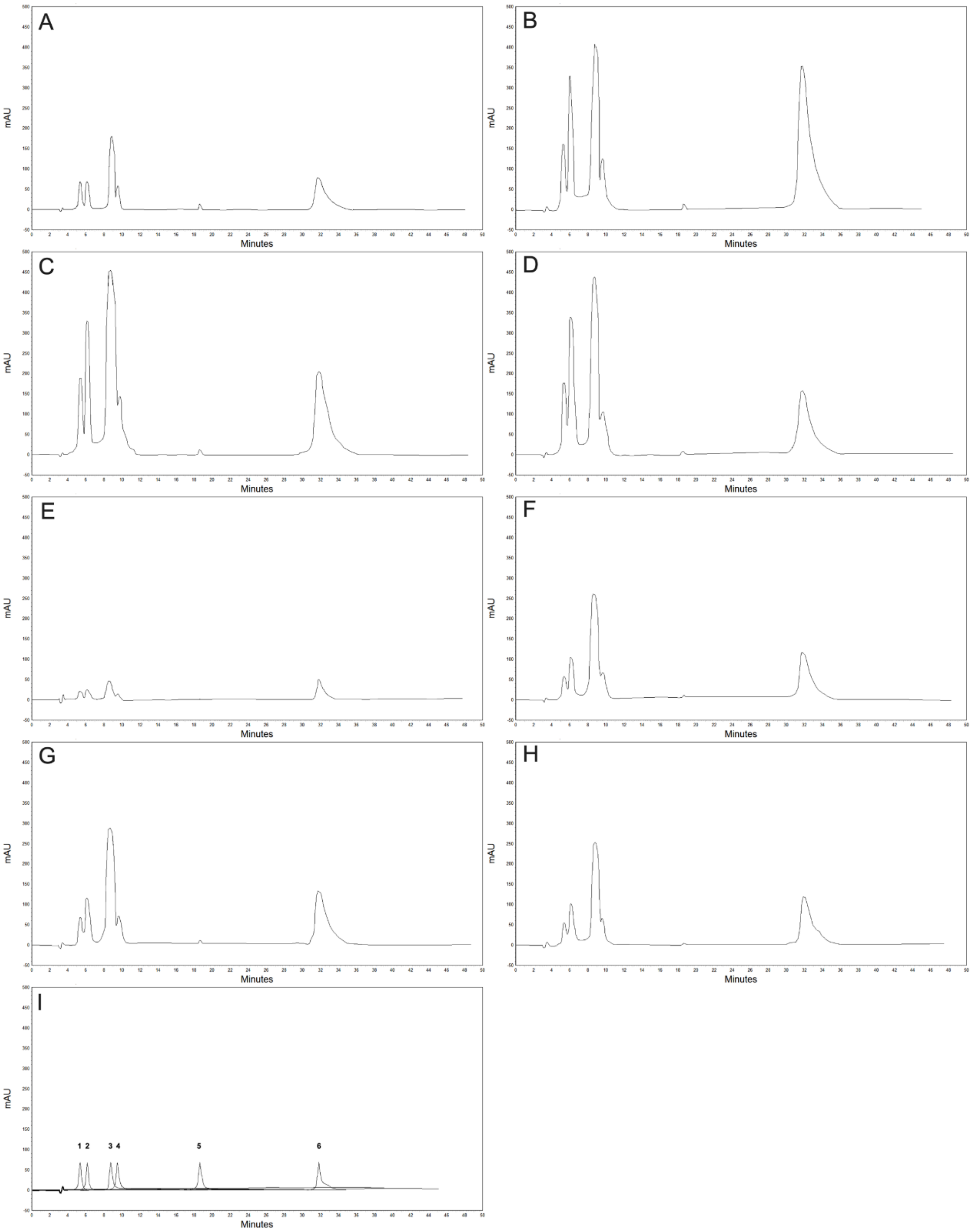

Supplementary Figure 10. The chromatogram figures of HPLC analysis. The chromatogram figures of **(A)** control plants and tobacco over-expressing **(B)** *LcLCYB*, **(C)** *LcLCYE,* and **(D)** *LcBCH* growing under normal condition; **(E)** control plants and tobacco over-expressing **(F)** *LcLCYB*, **(G)** *LcLCYE,* and **(H)** *LcBCH* growing under salt stress. The chromatogram figures of standard samples were shown in **(I)**, and peaks labeled with numbers were standard samples of **(1)** neoxanthin, **(2)** violaxanthin, **(3)** lutein, **(4)** zeaxanthin, **(5)** lycopene, and **(6)** β-carotene.

Supplementary Table 1. Physichemical properties and predicted subcellular localizations

Gene	Accession No.	Length of ORF (bp)	Number of amino acids (aa)	Molecular weight (Da)	pI of protein	Predicted Subcellular location
LcLCYB	KJ624406	1272	423	47807.6	8.06	chloroplast
LcLCYE	KJ143993	1749	582	64949.0	6.50	chloroplast
LcBCH	KF430643	939	312	34850.3	8.47	chloroplast

Supplementary Table 2. Information of the protein for the sequence analysis

Protein	Species	Accession number
AtBCH	*Arabidopsis thaliana*	NP_200070
CaBCH	*Capsicum annuum*	KAF3668875
CsaBCH	*Cucumis sativus*	XP_004140758
CsiBCH	*Citrus sinensis*	NP_001275830
EsBCH	*Eutrema salsugineum*	XP_006413280
GmBCH	*Glycine max*	NP_001242409
LsBCH	*Lactuca sativa*	XP_023769464
NtBCH	*Nicotiana tabacum*	NP_001313021
OsBCH	*Oryza sativa*	XP_015628773
SlBCH	*Solanum lycopersicum*	NP_001234348
StBCH	*Solanum tuberosum*	ADF28628
VvBCH	*Vitis vinifera*	RVW41947
ZmBCH	*Zea mays*	NP_001105865
AtLCYB	*Arabidopsis thaliana*	NP_187634
CaLCYB	*Capsicum annuum*	PHT71462
CsaLCYB	*Cucumis sativus*	XP_004150761
CsiLCYB	*Citrus sinensis*	AAM21152
EsLCYB	*Eutrema salsugineum*	XP_006407592
GmLCYB	*Glycine max*	XP_003554132
LsLCYB	*Lactuca sativa*	XP_023755637
NtLCYB	*Nicotiana tabacum*	NP_001311716
OsLCYB	*Oryza sativa*	XP_015627234
SlLCYB	*Solanum lycopersicum*	XP_010312096
StLCYB	*Solanum tuberosum*	XP_006351264
VvLCYB	*Vitis vinifera*	XP_002275769
ZmLCYB	*Zea mays*	NP_001169155
AtLCYE	*Arabidopsis thaliana*	NP_200513
CaLCYE	*Capsicum annuum*	PHT73568
CsaLCYE	*Cucumis sativus*	XP_004141172
CsiLCYE	*Citrus sinensis*	AAS48096
EsLCYE	*Eutrema salsugineum*	XP_006401252
GmLCYE	*Glycine max*	XP_003533775
LsLCYE	*Lactuca sativa*	XP_023756930
NtLCYE	*Nicotiana tabacum*	XP_016467034
OsLCYE	*Oryza sativa*	XP_015622198
SlLCYE	*Solanum lycopersicum*	NP_001234337
StLCYE	*Solanum tuberosum*	XP_006353544
VvLCYE	*Vitis vinifera*	RVW43956
ZmLCYE	*Zea mays*	AQK96213

Supplementary Table 3. Primers used for vector construction in this study

Gene	Primer sequences for vector construction (5'-3')
LcLCYB	F: ATCGGGATCCATGGATACTTTGTTGAAAACCCCAAATA
	R: ATCGGTCGACTTATTCTGAATCCTGTAACAAATTG
LcLCYE	F: ATCGGGATCCATGGAATGTGTTGGAGTTCAAAATTTTG
	R: ATCGGTCGACTTAAAATGTAAGATAAGTTCTTATC
LcBCH	F: ATCGGGATCCATGGCTGCCGGAATTTCAGGCATTG
	R: ATCGGTCGACTCATAATCCCTTAGAAATTTTAACT

Supplementary Table 4. Information of carotenoids analyzed with HPLC

Peak Number	Chemical Name	CAS Number	Retention Time (second)
1	neoxanthin	14660-91-4	322.37
2	violaxanthin	126-29-4	369.71
3	lutein	127-40-2	525.26
4	zeaxanthin	144-68-3	570.35
5	lycopene	502-65-8	1118.2
6	β-carotene	7235-40-7	1909.4

Supplementary Table 5. Primers used for Real-time PCR in this study

Gene	Primer Sequence (5'-3')	Accession number
NtActin	F: CCTGAGGTCCTTTTCCAACCA<br>R: GGATTCCGGCAGCTTCCATT	XM_016658252.1
NtIPI	F: AAGAGCATTGAAGAGGACGAG<br>R: AACAATGACCAATAACTCCAGGC	NM_001326211.1
NtGGPS	F: CTGTCAATCGAGCCTTAGATGC<br>R: AAACCCGCTTGCCTTCG	NM_001325671.1
NtPSY	F: TGTTGCTTTGTTGTGGGTTG<br>R: TTTGTCTCCCGCCTTTCA	NM_001325140.1
NtPDS	F: AGTCAGACTAAACTCACGAATAAA<br>R: CTCCCACTAGCTTCTCCAA	XM_016642616.1
NtZDS	F: GGGAGCTTGATTTCCGATTT<br>R: CCCGCACCACTGGACTAA	XM_016658599.1
NtCRTISO	F: GCAGGACCAGATTCAGCG<br>R: GATGGATAAAGGAGGTAGAGCC	NM_001325775.1
NtLCYB	F: AAGAGCATTGAAGAGGACGAG<br>R: TGAGGGATGAACCAGACCAG	NM_001324787.1
NtLCYE	F: GCTCTTGCTGCGGAGTC<br>R: GAATTGGATCGGCATCGT	NM_001325477.1
NtBCH	F: CTTGGCAAATGGAGGGTG<br>R: AAGAAGAGCAATGGCTGGAA	NM_001326092.1
NtVDE	F: CTGAAAGAGTGCAGGTTAGAGC<br>R: TGGAGACAGGCAACATTAGC	NM_001324805.1
NtZEP	F: CAACAGCAGAAACTGAGCCAT<br>R: AACAATGACCAATAACTCCAGGC	XM_016582459.1
NtNSY	F: AAGCCAAAGTAGGTGCCCAA<br>R: AACGGTAAGATGGAGAATGGCT	XM_016625496.1
NtAPX	F: GGAAGAGTTGGGAGTGGTGG<br>R: ACAGCTGCCAAATTCAAATCCT	NM_001324874.1
NtCAT	F: GTCTTGACAGGAAGGCGTGA<br>R: AACGGAAGACAGAGTAGCAGC	NM_001325412
NtP5CR	F: AAGAGCGTGGCACAATCACT<br>R: AGCTTTTCTCGAGTTTTCTTCTGC	XM_016610055.1
NtPOD	F: GGACAAATGGAGAGATTCGTTCAG<br>R: TGCTGACTTGGCCTACCAAC	XM_016632447.1
NtSOD	F: TCCTCCCCAGGTTCGGAATA<br>R: TGCGCTACATTTGGCAAGAG	XM_016631233.1
LcLCYB	F: ACATCCCTTGTAGCTCGTCCTGG<br>R: ACCATGCCAGCAGTACCACCA	KP262047.1
LcLCYE	F: AACGTTGGGCTCGTTGGGCCT<br>R: CTCCTCGTGCAGTAAATGGCGACT	KF768738.2

LcBCH	F: AGGCTGGCGGAAAAATTGGCGA<br>R: GCAGCGCCAAACGAGAGAGCGA	KF430643.1
